# Blood and Site of Disease Inflammatory Profiles Differ in Patients With Pericardial Tuberculosis and Human Immunodeficiency Virus Type 1

**DOI:** 10.1093/ofid/ofad128

**Published:** 2023-03-09

**Authors:** Hygon Mutavhatsindi, Elsa Du Bruyn, Sheena Ruzive, Patrick Howlett, Maddalena Cerrone, Alan Sher, Katrin D Mayer-Barber, Daniel L Barber, Mpiko Ntsekhe, Robert J Wilkinson, Catherine Riou

**Affiliations:** Wellcome Centre for Infectious Disease Research in Africa, Institute of Infectious Disease and Molecular Medicine, University of Cape Town, Observatory, South Africa; Division of Medical Virology, Department of Pathology, University of Cape Town, Observatory, South Africa; Wellcome Centre for Infectious Disease Research in Africa, Institute of Infectious Disease and Molecular Medicine, University of Cape Town, Observatory, South Africa; Wellcome Centre for Infectious Disease Research in Africa, Institute of Infectious Disease and Molecular Medicine, University of Cape Town, Observatory, South Africa; Wellcome Centre for Infectious Disease Research in Africa, Institute of Infectious Disease and Molecular Medicine, University of Cape Town, Observatory, South Africa; Wellcome Centre for Infectious Disease Research in Africa, Institute of Infectious Disease and Molecular Medicine, University of Cape Town, Observatory, South Africa; Department of Infectious Diseases, Imperial College London, London, United Kingdom; The Francis Crick Institute, London, United Kingdom; Immunobiology Section, Laboratory of Parasitic Diseases, National Institute of Allergy and Infectious Diseases, National Institutes of Health, Bethesda, Maryland, USA; Inflammation and Innate Immunity Unit, Laboratory of Clinical Immunology and Microbiology, National Institute of Allergy and Infectious Diseases, National Institutes of Health, Bethesda, Maryland, USA; T Lymphocyte Biology Section, Laboratory of Parasitic Diseases, National Institute of Allergy and Infectious Diseases, National Institutes of Health, Bethesda, Maryland, USA; Wellcome Centre for Infectious Disease Research in Africa, Institute of Infectious Disease and Molecular Medicine, University of Cape Town, Observatory, South Africa; Department of Medicine, University of Cape Town, Observatory, South Africa; Division of Cardiology, Department of Medicine, University of Cape Town, Observatory, South Africa; Wellcome Centre for Infectious Disease Research in Africa, Institute of Infectious Disease and Molecular Medicine, University of Cape Town, Observatory, South Africa; Department of Infectious Diseases, Imperial College London, London, United Kingdom; The Francis Crick Institute, London, United Kingdom; Department of Medicine, University of Cape Town, Observatory, South Africa; Division of Cardiology, Department of Medicine, University of Cape Town, Observatory, South Africa; Wellcome Centre for Infectious Disease Research in Africa, Institute of Infectious Disease and Molecular Medicine, University of Cape Town, Observatory, South Africa; Division of Medical Virology, Department of Pathology, University of Cape Town, Observatory, South Africa

**Keywords:** diagnosis, inflammatory profile, pericardial tuberculosis, site of disease, treatment response

## Abstract

**Background:**

To better understand the pathogenesis of pericardial tuberculosis (PCTB), we sought to characterize the systemic inflammatory profile in people with human immunodeficiency virus type 1 (HIV-1) with latent TB infection (LTBI), pulmonary TB (PTB), or PCTB.

**Methods:**

Using Luminex, we measured the concentration of 39 analytes in pericardial fluid (PCF) and paired plasma from 18 PCTB participants, and plasma from 16 LTBI and 20 PTB participants. Follow-up plasma samples were also obtained from PTB and PCTB participants. HLA-DR expression on *Mycobacterium tuberculosis*–specific CD4 T cells was measured in baseline samples using flow cytometry.

**Results:**

Assessment of the overall systemic inflammatory profile by principal component analysis showed that the inflammatory profile of active TB participants was distinct from the LTBI group, while PTB patients could not be distinguished from those with PCTB. When comparing the inflammatory profile between PCF and paired blood, we found that the concentrations of most analytes (25/39) were elevated at site of disease. However, the inflammatory profile in PCF partially mirrored inflammatory events in the blood. After TB treatment completion, the overall plasma inflammatory profile reverted to that observed in the LTBI group. Lastly, HLA-DR expression showed the best performance for TB diagnosis compared to previously described biosignatures built from soluble markers.

**Conclusions:**

Our results show that the inflammatory profile in blood was comparable between PTB and PCTB. However, at the site of infection (PCF), inflammation was significantly elevated compared to blood. Additionally, our data emphasize the potential role of HLA-DR expression as a biomarker for TB diagnosis.

Tuberculosis (TB) is the leading cause of death among people with human immunodeficiency virus type 1 (HIV-1) [1]. Moreover, 15%–20% of all TB cases in developing countries are accounted for by extrapulmonary TB (EPTB) [2, 3], which disproportionately affects immunocompromised patients [4, 5]. Pericardial TB (PCTB), a severe form of EPTB, is the most common cause of pericarditis in TB-endemic countries in Africa and Asia [6, 7]. PCTB-related morbidity is significant, with mortality (which generally occurs early in the onset of the disease) as high as 26% and increasing to approximately 40% in cohorts of predominantly people with HIV (PWH) [8].

HIV impairs both innate and adaptive immune responses, with the most obvious immune defect being a progressive reduction in absolute CD4^+^ T-cell numbers and systemic hyperactivation [9]. HIV-1 has also been shown to alter the balance of *Mycobacterium tuberculosis* (*Mtb*)–specific T-helper subsets, through the reduction of Th17 cells and T regulatory (Treg) cells [10–12], suggesting that HIV shifts *Mtb*-specific responses toward a more pathogenic/inflammatory profile [10].

Pulmonary TB (PTB)–induced systemic inflammation has been studied extensively showing high concentrations of acute phase proteins and proinflammatory cytokines including C-reactive protein (CRP), serum amyloid P component (SAP), interferon gamma (IFN-γ), interferon γ–induced protein 10 (IP-10), chemokine (C-C motif) ligand 1 (CCL1), and tumor necrosis factor alpha (TNF-α) in serum/plasma of active TB participants in comparison to other respiratory diseases, latent tuberculosis infection (LTBI), or healthy controls [13–16]. Furthermore, in patients with PTB admitted to intensive care units, serum levels of inflammatory factors such as interleukin (IL)–1, IL-6, IL-10, IL-12, and IL-4 are upregulated compared to healthy controls [17]. Based on these results, several host inflammatory marker signatures have been proposed as biomarkers for TB diagnosis and the monitoring of treatment response, with superior performance compared to smear microscopy [13, 14, 18, 19].

However, the influence of HIV-1 coinfection on the immune response to *Mtb* in the context of PTB and EPTB remains poorly understood. Moreover, studies assessing immune responses at site of disease are scarce [20–22]. These studies reported higher levels of cytokines/chemokines at the site of disease in comparison to paired peripheral blood with exception of a few analytes, such as IFN-γ, IL-1β, and IL-8, which were reported to be significantly higher in peripheral blood instead [20–22]. Thus, in the current study, we measured 39 soluble markers in blood and at site of disease (pericardial fluid [PCF]) to (1) compare the systemic cytokine environment between pulmonary and pericardial TB (PCTB) patients coinfected with HIV-1; (2) define the relationship between HIV viral load and the inflammatory profiles; (3) define whether peripheral inflammation signatures mirror those at site of infection; (4) assess the impact of TB treatment on systemic inflammation; and (5) evaluate the performance of previously described blood-based biomarkers to discriminate latent from active TB.

## METHODS

### Study Population

Participants were recruited between June 2017 and April 2019. Patients with suspected PCTB were recruited from the Groote Schuur Hospital Cardiology Unit. Only adults (≥18 years of age) who were undergoing pericardiocentesis as part of the routine management of their pericardial effusion were included. The PCTB group included patients with either definite (*Mtb* culture positive in pericardial fluid [PCF], n = 9) or probable PCTB (n = 9). Probable PCTB was defined based on evidence of pericarditis with microbiologic confirmation of *Mtb* infection elsewhere in the body and/or an exudative, lymphocyte-predominant pericardial effusion with elevated adenosine deaminase (≥35 U/L), according to Mayosi et al [23]. Only 3 PCTB patients were HIV negative and 2 PCTB patients with HIV were on antiretroviral therapy (ART) at the time of enrollment. Paired PCF and blood were collected at the same time for PCTB patients.

Participants with LTBI or PTB were recruited at the Ubuntu Clinic, Site B, a community health clinic located in Khayelitsha, Cape Town, South Africa. Patients included in the PTB group (n = 20) were all ART-naive PWH, tested sputum Xpert MTB/RIF (Xpert, Cepheid, Sunnyvale, California) positive, and had clinical symptoms and/or radiographic evidence of TB. All were infected by drug-sensitive isolates of *Mtb* and had received no more than 1 dose of antitubercular treatment at the time of baseline blood sampling. Participants included in the LTBI group (n = 16) were all PWH, asymptomatic, had a positive IFN-γ release assay (QuantiFERON-TB Gold In-Tube, Qiagen, Hilden, Germany), tested sputum Xpert MTB/RIF negative, and exhibited no clinical evidence of active TB. Seventy-five percent of the LTBI participants were ART naive. None of the participants included in the study reported a previous history of TB.

Sputum and PCF *Mtb* culture, CD4 cell count, and HIV viral load (VL) were performed by the South African National Health Laboratory Services. Patients with active TB (PTB or PCTB) were followed up over the duration of their antitubercular treatment and additional blood draws were performed at week 6 for PCTB, week 8 for PTB, and week 24 for both disease groups. All participants were adults (age ≥18 years).

### Patient Consent Statement

All participants provided written informed consent. The study design was approved by the University of Cape Town Human Research Ethics Committee (050/2015 and 271/2019).

### Pericardial Fluid, Blood Collection, and Whole Blood Assay

Pericardial fluid was obtained at the time of pericardiocentesis, placed in sterile Falcon tubes, and transported to the laboratory at 4°C. Blood was collected in sodium heparin tubes and processed within 3 hours of collection. The whole blood or whole PCF assays were adapted from the protocol described by Hanekom et al [24]. In brief, 0.5 mL of whole blood or 1 mL of whole PCF was stimulated with a pool of 300 *Mtb*-derived peptides (Mtb300, 2 μg/mL) [25] at 37°C for 5 hours in the presence of the co-stimulatory antibodies anti-CD28 and anti-CD49d (1 μg/mL each; BD Biosciences, San Jose, California) and brefeldin A (10 μg/mL; Sigma-Aldrich, St Louis, Missouri). Unstimulated cells were incubated with co-stimulatory antibodies and brefeldin A only. Red blood cells were then lysed in a 150 mM NH_4_Cl, 10 mM KHCO_3_, and 1 mM Na_4_ ethylenediaminetetraacetic acid solution. Cells were stained with a Live/Dead near-infrared dye (Invitrogen, Carlsbad, California) and fixed using a transcription factor fixation buffer (eBioscience, San Diego, California), cryopreserved in freezing media (50% fetal calf serum, 40% RPMI, and 10% dimethyl sulfoxide) and stored in liquid nitrogen until use. Mtb300 was used for T-cell stimulation as it is a comprehensive “megapool” of *Mtb* peptides that captures a large fraction of the *Mtb*-reactive T-cell response. Thus, high frequency of *Mtb*-responding cells will confer a better accuracy to define the activation profile on those cells [26].

### Cell Staining and Flow Cytometry

Cryopreserved cells were thawed, washed, and permeabilized with a transcription factor perm/wash buffer (eBioscience). Cells were then stained at room temperature for 45 minutes with the following antibodies: CD3 BV650 (OKT3; BioLegend, San Diego, California), CD4 BV785 (OKT4; BioLegend), CD8 BV510 (RPA-T8; BioLegend), HLA-DR BV605 (L243; BioLegend), IFN-γ BV711 (4S.B3; BioLegend), TNF-α PE-Cy7 (Mab11; BioLegend eBioscience), and IL-2 PE/Dazzle (MQ1-17H12; BioLegend). Samples were acquired on a BD LSR-II and analyzed using FlowJo (version 10.8.1, FlowJo, Ashland, Oregon). The gating strategy is presented in Supplementary Figure 1. A positive cytokine response was defined as at least twice the background of unstimulated cells. To define the phenotype of Mtb300-specific CD4 T cells, a cutoff of 30 events was used [27].

### Luminex Multiplex Immunoassay

Using Luminex technology, we measured the levels of 39 analytes using antibodies supplied by Merck Millipore (Billerica, Massachusetts) and R&D Systems (Minneapolis, Minnesota). The analytes measured were identified in literature with potential for TB diagnosis, monitoring of TB treatment response, or fibrosis-related analytes. The analytes measured included granzyme B, IL-2, IL-8, IL-12p40, macrophage colony-stimulating factor (M-CSF), TNF-α, transforming growth factor beta (TGF-β), complement component 3 (C3), complement component 4 (C4), CRP, SAP, IL-22, Galectin-3, intercellular adhesion molecule 1 (ICAM-1), neural cell adhesion molecule 1, granulocyte colony-stimulating factor (G-CSF), IFN-γ, IL-6, IL-10, IL-27, vascular endothelial growth factor (VEGF), monokine induced by gamma (MIG), monocyte chemoattractant protein 2 (MCP-2), granulocyte chemoattractant protein 2, chemokine (C-X-C motif) ligand 11 (CXCL11), macrophage inflammatory protein 1 beta (MIP-1β), CCL1, IP-10, cluster of differentiation 163 (CD163), interleukin 6 receptor alpha (IL-6Rα), cluster of differentiation 30 (CD30), interleukin 2 receptor alpha (IL-2Rα), apolipoprotein A-I (ApoA-I), apolipoprotein C-III (Apo-CIII), oncostatin M (OSM), interleukin 33 receptor (IL-33R), osteopontin (OPN), platelet-derived growth factor BB, and thrombomodulin. All samples were evaluated undiluted or diluted according to the manufacturer's recommendations. Samples were randomized to assay plates with the experimenter blinded to sample data. All assays were performed and read at University of Cape Town on the Bio-Plex platform (Bio-Rad), with the Bio-Plex Manager Software (version 6.1) used for bead acquisition and analysis.

### Statistical Analyses

Statistical tests were performed in Prism (version 9.1.3, GraphPad Software, San Diego, California). Nonparametric tests were used for all comparisons. The Kruskal-Wallis test with Dunn multiple comparison test was used for multiple comparisons, the Spearman rank test for correlation, and the Mann-Whitney and Wilcoxon matched pairs test for unmatched and paired samples, respectively. When the measured analyte was below the limit of detection in >20% of the samples (ie, M-CSF and IL-10), the analyte was not included in the correlation with plasma HIV VL and HLA-DR expression on *Mtb*-specific CD4 T cells. Unsupervised hierarchical clustering analysis (HCA, Ward method) and principal component analysis (PCA) were carried out in JMP (version 16.0.0; SAS Institute, Cary, North Carolina). For HCA and PCA, the min-max normalization method (i.e., feature scaling, analyte value – min / max – min) was used to scale data in the 0 to 1 range. The predictive abilities of combinations of analytes were investigated by general discriminant analysis in JMP. The diagnostic ability of HLA-DR expression on *Mtb*-specific CD4 T cells was assessed by receiver operating characteristic (ROC) curve analysis. Optimal cutoff values and associated sensitivity and specificity were determined based on the Youden index [28]. Analyte network analysis was performed using Gephi (version 0.9.2; University of Technology of Compiègne, Compiègne, France). A *P* value of <.05 was set as the significance threshold following Benjamini-Hochberg multiple testing correction [29] with a false discovery rate of 5%.

## RESULTS

### Study Population

The clinical characteristics of participants are presented in Table 1. Median age was comparable between the 3 groups. LTBI participants had a lower plasma HIV-1 VL compared to the PCTB and PTB groups (median log_10_ VL: 3.28 vs 4.68 and 4.79 copies/mL, respectively). Aviremic participants represented 25%, 13.33%, and 0% of the LTBI, PCTB, and PTB groups, respectively; HIV-1–unsuppressed (i.e., log_10_ VL >3) individuals represented 56.25%, 73.33%, and 85% of the LTBI, PCTB, and PTB groups, respectively. As expected, LTBI participants were also characterized by higher absolute CD4 count compared to the PCTB and PTB groups (median CD4: 409 vs 141 and 176 cells/µL, respectively). Approximately one-third (5/16 [31.25%]) of LTBI participants had a CD4 count >500 cells/µL, while all PCTB and PTB patients had a CD4 count <500 cells/µL.

**Table 1.** Clinical Characteristics of Study Participants

Characteristic	PCTB	PTB	LTBI
No.	18	20	16
Age, y^a^	36 (29–44)	39 (32–45)	37 (32–41)
Sex, female/male	8/10	8/12	16/0
HIV-1 status, negative/positive	3/15	0/20	0/16
CD4 count, cells/µL^a^	141 (61–195.3)	176 (107–246)	409 (264–524)
Log_10_ HIV-1 VL, mRNA copies/mL^a^	4.68 (2.903–5.278)	4.79 (4.23–5.11)	3.28 (1.44–4.18)
*Mtb* culture positive, No. (%)	9/16 (56.2) in PCF^b^	19 (95) in sputum	0 (0) in sputum

Abbreviations: HIV-1, human immunodeficiency virus type 1; LTBI, latent TB infection; mRNA, messenger RNA; *Mtb*, *Mycobacterium tuberculosis*; PCF, pericardial fluid; PCTB, pericardial tuberculosis; PTB, pulmonary tuberculosis; VL, viral load.

a Median (interquartile range).

b *Mtb* culture data were not available for 2 patients with PCTB.

### Comparison of the Systemic Inflammatory Profile Between LTBI, PTB, and PCTB

**Figure 1.**
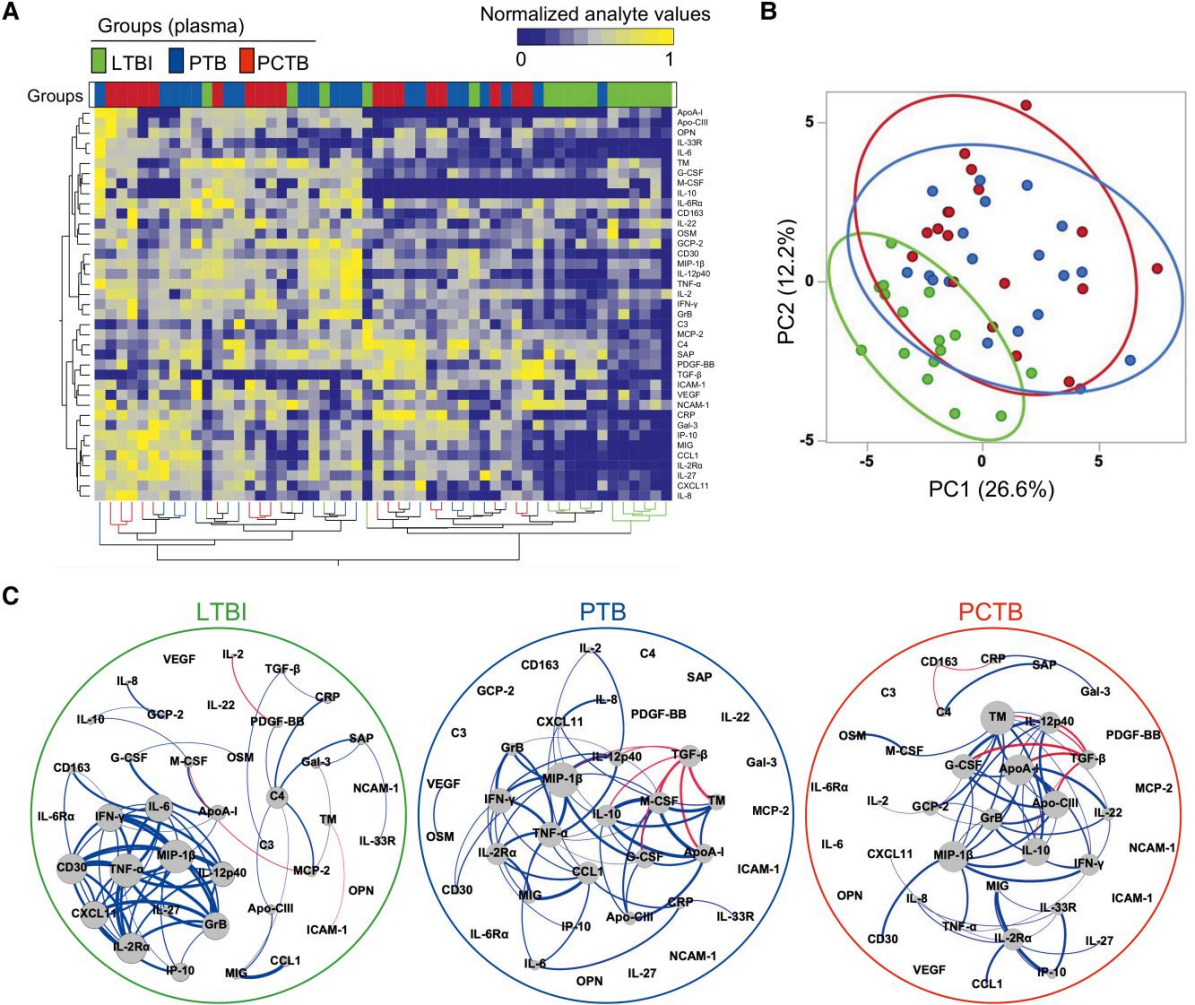
Analyte profiles in the different tuberculosis (TB) groups at baseline. *A*, Non-supervised 2-way hierarchical cluster analysis (Ward method) was employed to evaluate the TB groups using the 39 measured analytes (see Materials and Methods for expansions of abbreviations of analytes). TB status (pericardial TB [PCTB] in red, pulmonary TB [PTB] in blue, and latent TB infection [LTBI] in green) of each patient is indicated at the top of the dendrogram. Data are depicted as a heatmap colored from minimum to maximum normalized values for each marker. *B*, Principal component analysis on correlations based on the 39 analytes was used to explain the variance of the data distribution in the cohort. Each dot represents a participant. The 2 axes represent principal components 1 (PC1) and 2 (PC2). Their contribution to the total data variance is shown as a percentage. *C*, Analyte network analysis (Fruchterman-Reingold algorithm) in plasma of LTBI, PTB, and PCTB participants. Size of nodes indicates the number of connections. Size of edges indicates the Spearman *r* value (only *r* >0.6 was included). Blue lines: positive correlation. Red lines: negative correlation.

Plasma levels of 39 analytes, including cytokines, chemokines, apolipoproteins, chemokine and protein receptors, and fibrosis-related analytes, were measured in all participants (the complete list of measured analytes is presented in the Materials and Methods). Assessing the overall systemic inflammatory profile using unsupervised hierarchical clustering (Figure 1*A*) and PCA (Figure 1*B*), we showed an evident separation between LTBI and active TB participants (PCTB and PTB), driven by elevated levels of most of the measured inflammatory markers. However, there was no noticeable separation between the PCTB and PTB groups, suggesting comparable systemic inflammation in these groups. Individual analysis of measured analytes showed that 15 markers were significantly higher in both PTB and PCTB compared to the LTBI group, including innate-related inflammation markers (eg, IL-6, TNF-α, and IL-8), acute phase protein (CRP), and chemokines (CCL1, MIG, IP-10, and CXCL11). VEGF also showed a similar profile, with the *P* value between LTBI and PTB being borderline significant (*P* = .0503) (Supplementary Figure 2 and Supplementary Table 1). IL-6Rα and G-CSF were the only markers that were observed to be differentially expressed between PTB and PCTB (Supplementary Figure 2 and Supplementary Table 1), highlighting similarities between the different clinical forms of TB. Only 1 marker, OPN, showed increased expression levels, only in the PCTB group compared to LTBI (*P* = .0063); no significant difference was observed for the PTB group (*P* = .374) (Supplementary Figure 2 and Supplementary Table 1). Elevated OPN levels have been associated with severe TB [30]. Next, we defined the interplay between markers, using network analysis (Fruchterman-Reingold algorithm, Figure 1*C*). In LTBI participants, TNF-α and MIP-1β were the most central nodes, showing the most connections (positive associations) with other analytes. In active TB patients (both PTB and PCTB), the network structure was substantially altered; while MIP-1β remained a predominant node, TGF-β emerged as a new influential node, with multiple negative associations with analytes such as IL-12p40, ApoA-I, and G-CSF (Figure 1*C*). Overall, these results illustrate that active TB disease significantly increases systemic inflammation and that PCTB and PTB participants share similar inflammatory signatures.

### Relationship Between Inflammatory Profile and HIV-1 VL

**Figure 2.**
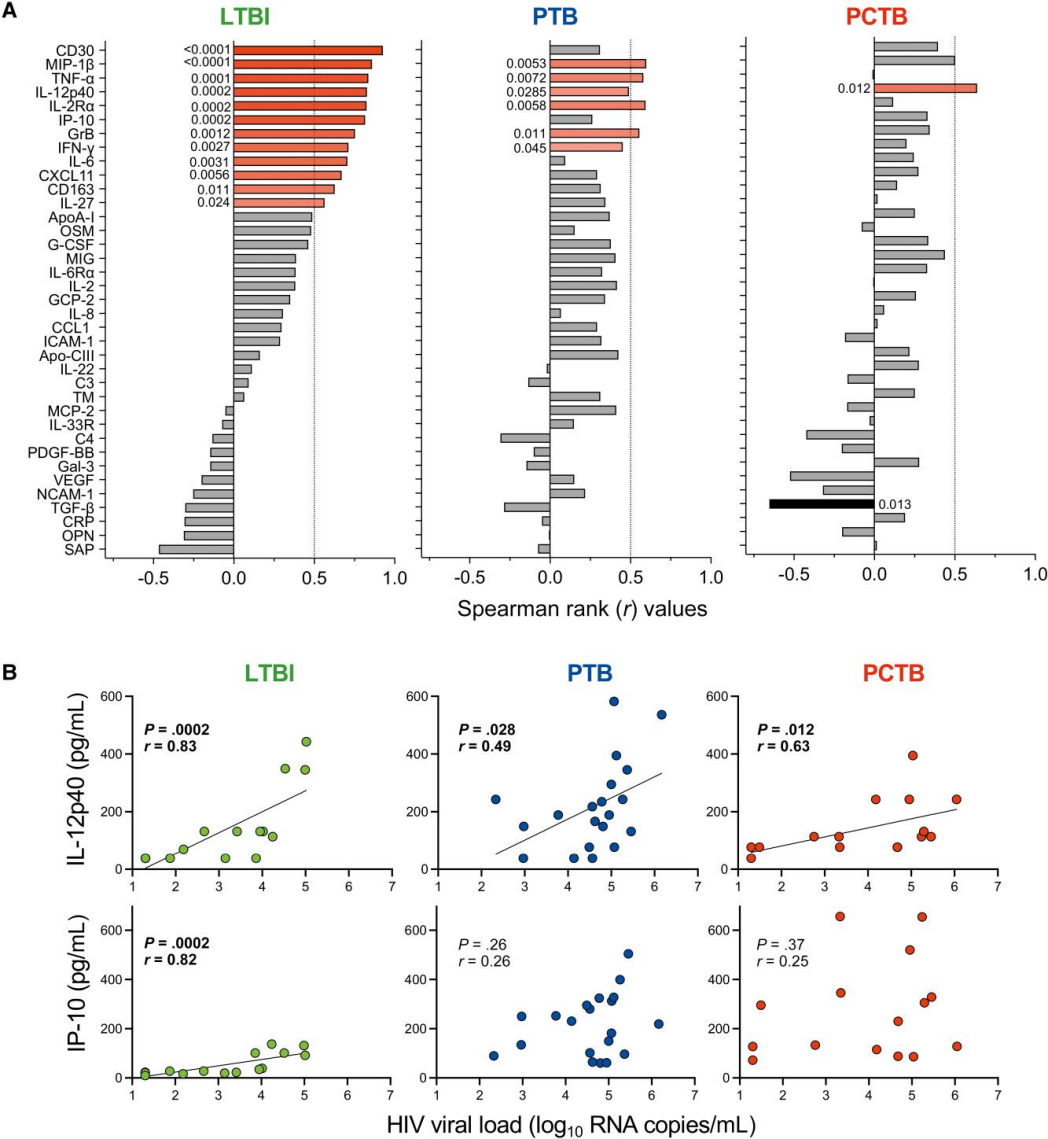
Univariate associations between human immunodeficiency virus type 1 (HIV-1) viral load (VL) and analyte concentrations in the different tuberculosis (TB) groups. *A*, Spearman rank values of the univariate correlation between each analyte (see Materials and Methods for expansions of abbreviations of analytes) and the HIV-1 VL in plasma samples of participants with latent TB infection (LTBI), pulmonary TB (PTB), and pericardial TB (PCTB). Red bars indicate positive correlations, Black bars indicate negative correlations, and gray bars indicate nonsignificant correlations. *B*, Examples of IL-12p40 (maintained relationship between the TB groups) and IP-10 (disrupted relationship between the TB groups). The line indicates linear regression for statistically significant correlations.

To examine the interplay between HIV-1 VL and cytokine profile, we defined the associations between cytokine concentrations and HIV-1 VL in plasma. Of the 39 measured analytes, 12 markers positively associated with HIV-1 VL in the LTBI group (Figure 2*A*). Several of those have been previously reported as HIV-1–associated systemic inflammation markers, including IL-2Rα [31], CXCL11 [32], IL-6 [33], IFN-γ [34], IP-10 [35], TNF-α [35], and CD30 [36]. In both the PTB and PCTB groups, most of these correlations were disrupted with 6 analytes correlating with HIV-1 VL in the PTB group and only 1 in the PCTB group (Figure 2*A*). The only cytokine that maintained significant correlation with HIV-1 VL in all groups was IL-12p40, albeit the correlation strength was weaker in the disease groups (*r* = 0.83, *P* = .0002 vs *r* = 0.49, *P* = .028 in the PTB group and *r* = 0.63, *P* = .012 in the PCTB group) (Figure 2*B*). IP-10 concentration only showed a significantly positive correlation with HIV-1 VL in the LTBI group (*r* = 0.82, *P* = .0002) and was largely disrupted in both the PTB and PCTB groups (*r* = 0.29, *P* = .26 and *r* = 0.25, *P* = .37, respectively) (Figure 2*B*). No negative associations were observed in the LTBI and PTB groups; however, TGF-β showed a strong negative association with HIV-1 VL in the PCTB group (*r* = −0.65, *P* = .0133) (Figure 2*A*). These findings suggest that active TB disease disrupts HIV-1–associated systemic inflammation. It is likely that the synergy between HIV and *Mtb* infection exacerbate inflammation, where metabolic changes are altered compared to HIV infection or TB alone.

### Profile of Soluble Markers in Plasma Compared to Pericardial Fluid

**Figure 3.**
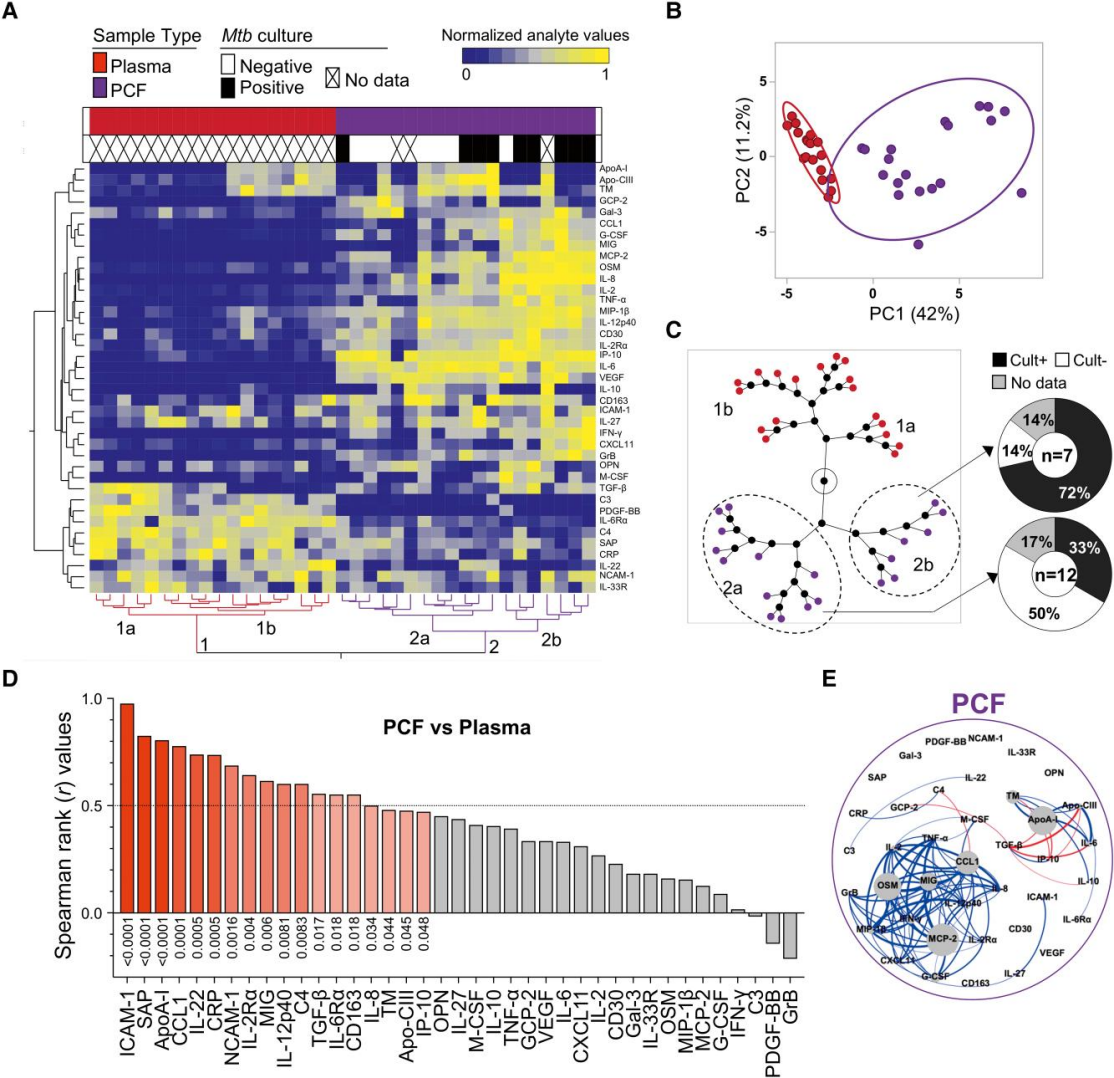
Analyte profiles in peripheral blood (plasma) and site of disease (pericardial fluid [PCF]) in participants with pericardial TB (PCTB). *A*, Nonsupervised 2-way hierarchical cluster analysis (HCA, Ward method) was employed to evaluate the 2 sites using the 39 analytes. The sample type and *Mycobacterium tuberculosis* (*Mtb*) culture results (PCF in purple; plasma in red; *Mtb* culture negative in white and positive in black) of each patient is indicated at the top of the dendrogram. Data are depicted as a heatmap colored from minimum to maximum normalized values detected for each marker. *B*, Principal component analysis on correlations based on the 39 analytes was used to explain the variance of the data distribution in the subgroup. Each dot represents a participant. The 2 axes represent principal components 1 (PC1) and 2 (PC2). Their contribution to the total data variance is shown as a percentage. *C*, Constellation plot-cluster analysis based on all measured analytes. Each dot represents a participant and is color-coded according to sample type. Each cluster obtained for the HCA is identified by a number. *D*, Pairwise correlation of the 39 analytes (see Materials and Methods for expansions of abbreviations of analytes). Red bars indicate a positive correlation, black bars indicate a negative correlation, and gray bars indicate a nonsignificant correlation. *E*, Analyte network analysis in PCF of participants with PCTB. Size of nodes indicates the number of connections. Size of edges indicates the Spearman *r* (only *r* >0.6 was included). Blue lines: positive correlation. Red lines: negative correlation.

To better understand compartmentalization, we compared the profiles of expression of the 39 measured analytes in plasma and PCF from PCTB participants, using HCA and PCA (Figure 3*A* and 3*B*). There was a clear separation between sample types, where PC1 accounted for 42% and PC2 11.2% of the variance (Figure 3*B*). Furthermore, visualizing sample clustering using a constellation plot, we observed that cluster 2 (comprised of PCF samples) was divided into 2 distinct subclusters, where cluster 2b was enriched in participants who were PCF culture positive (5/7 [72%]) compared to patients included in cluster 2a (4/12 [33%]) (Figure 3*C*). However, looking at individual analytes, we did not find a significant difference between PCF culture-negative and PCF culture-positive samples (data not shown).

Univariate analysis of analytes showed that the concentrations of 25 of the 39 measured analytes were significantly higher in PCF in comparison to paired plasma samples, only 9 of 39 were significantly higher in plasma compared to PCF, and 5 of 39 showed no significant difference in expression between the 2 sample types after correction of the *P* values for multiple testing (Supplementary Figure 3 and Supplementary Table 2).

To better understand the relationship between peripheral and site of disease inflammation, pairwise comparisons (plasma vs PCF) were assessed. Significant positive correlations were observed for 18 of the 39 analytes (with *r* and *P* ranging from 0.98 to 0.47 and <.0001 to .048, respectively); the highest Spearman rank *r* values for significant positive correlations were observed for ICAM-1, SAP, and ApoA-I (Figure 3*D*). A summarized representation of the associations between plasma and PCF for each analyte is shown in Figure 3*D* and individual correlation plots of all the significant associations are presented in Supplementary Figure 4. We then defined the interplay between markers in PCF, using network analysis (Fruchterman-Reingold algorithm, Figure 3*E*). OSM, MCP-2, and ApoA-I were the most central nodes, with OSM and MCP-2 showing positive associations with other analytes, whereas ApoA-I showed mostly negative associations with analytes such as TGF-β, IP-10, and Apo-CIII (Figure 3*E*). Overall, these results show that inflammatory response at site of disease was greater than in blood. However, the inflammatory profile in PCF partially mirrored inflammatory events in blood.

### Associations Between Systemic Inflammation and the Activation of *Mtb*-Specific CD4^+^ T Cells in Blood and at Site of Disease

**Figure 4.**
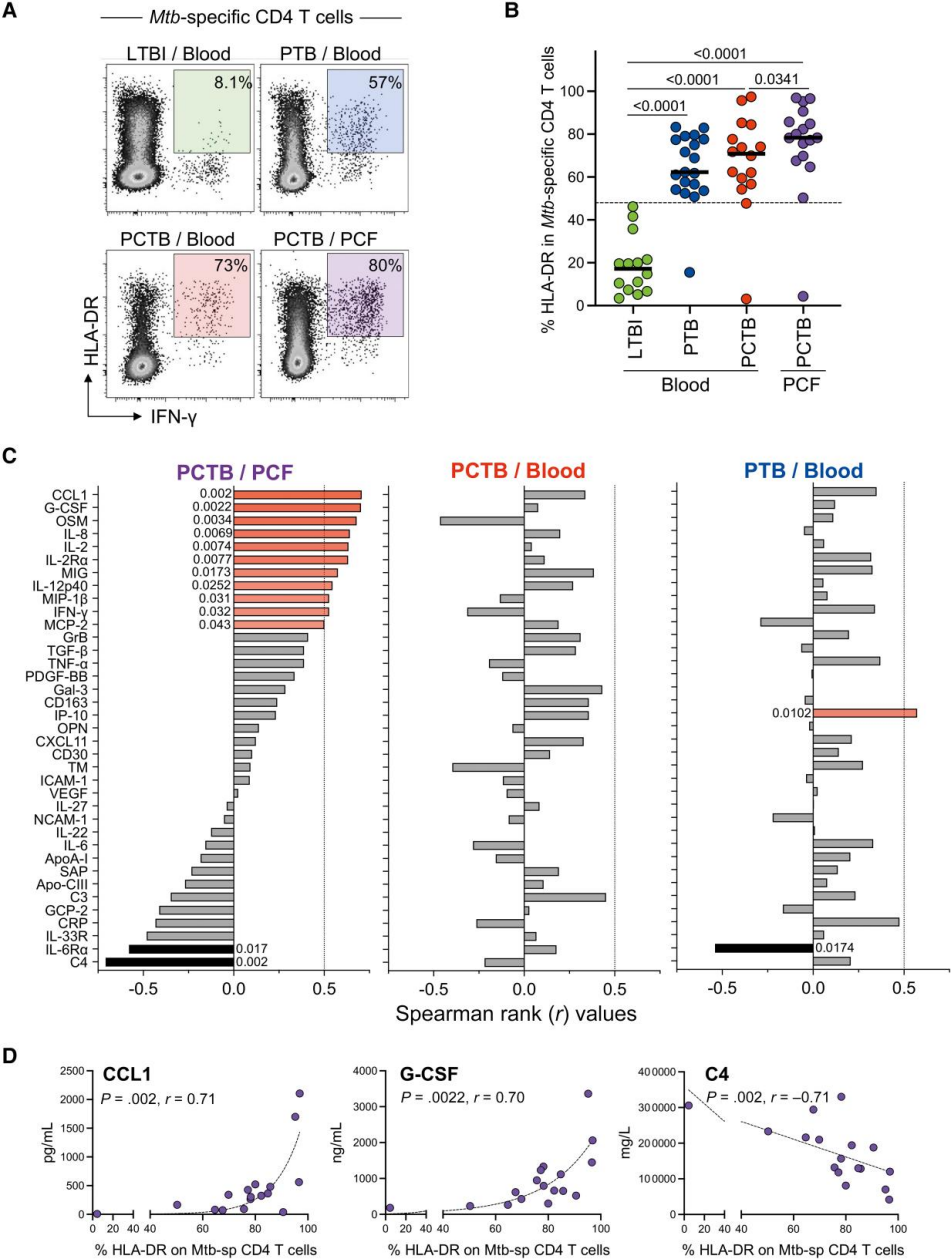
Univariate associations between HLA-DR expression on *Mycobacterium tuberculosis* (*Mtb*)–specific CD4 T cells and analyte concentrations in the different tuberculosis (TB) groups. *A*, Representative flow cytometry plots of the expression of HLA-DR on *Mtb*-specific CD4 T cells. *B*, Expression of HLA-DR on *Mtb*-specific CD4 T cells in response to Mtb300. *C*, Spearman rank values of the univariate correlation between each analyte (see Materials and Methods for expansions of abbreviations of analytes) and between *Mtb*-specific CD4 T-cell activation (HLA-DR) level at the site of disease (pericardial fluid [PCF]) in participants with pericardial TB (PCTB), in blood of participants with PCTB and pulmonary TB (PTB), respectively. Red bars indicate a positive correlation, black bars indicate a negative correlation, and the gray bars indicate nonsignificant correlation. *D*, Representative graphs showing the positive (CCL1 and G-CSF) and negative (C4) correlation to HLA-DR frequency at the site of disease (PCF). Statistical comparisons were performed using a Kruskal-Wallis test, adjusted for multiple comparisons (Dunn test) for blood latent TB infection (LTBI) vs PTB vs PCTB, Wilcoxon test for blood PCTB vs PCF PCTB, and the Mann-Whitney test to compare blood LTBI and PCF PCTB.

HLA-DR expression on peripheral *Mtb*-specific CD4^+^ T cells has been shown to discriminate latent from active TB infection [37–39]. To better understand the relationship between inflammation and T-cell activation, we measured the expression of HLA-DR on *Mtb*-specific CD4^+^ T cells in blood from LTBI, PTB, and PCTB participants, and PCF from PCTB participants. As expected, HLA-DR expression on peripheral *Mtb*-specific CD4^+^ T cells was significantly higher in the active TB groups (PTB and PCTB) compared to LTBI (median, 62.30% and 70.85% vs 17.20%, respectively, *P* > .0001). Moreover, HLA-DR expression on *Mtb*-specific CD4^+^ T cells in PCF was significantly higher compared to blood in the PCTB group (medians, 78.30% vs 69.90%, respectively, *P* = .0341) (Figure 4*A* and 4*B*). We then assessed the association of HLA-DR expression on *Mtb*-specific CD4 T cells and the concentrations of each measured analyte at the site of disease (PCF) and in blood from PCTB participants as well as blood from PTB participants (Figure 4*C*). At disease site, we observed positive associations between HLA-DR expression on *Mtb*-specific CD4 T cells and 11 of the measured analytes, including CCL1 and G-CSF (strong correlations with *r* value >0.7), and 9 analytes (OSM, IL-8, IL-2, IL-2Rα, MIG, IL-12p40, MIP-1β, IFN-γ, and MCP-2) with moderate correlations (*r* value between 0.5 and 0.7). Negative associations were observed with C4 (*r* = −0.71, *P* = .002) and IL-6Rα (*r* = −0.54, *P* = .017) (Figure 4*D*). None of these associations were observed in peripheral blood (Figure 4*C*). In PTB participants, HLA-DR expression on peripheral *Mtb*-specific CD4^+^ T cells was associated with only 2 analytes, namely IP-10 (*r* = 0.57, *P* = .0102) and IL-6Rα (*r* = −0.54, *P* = .0174) (Figure 4*C*). These data suggest a coordinated and compartmentalized immune response at the disease site.

### Impact of TB Treatment on the Inflammatory Profile in Plasma

**Figure 5.**
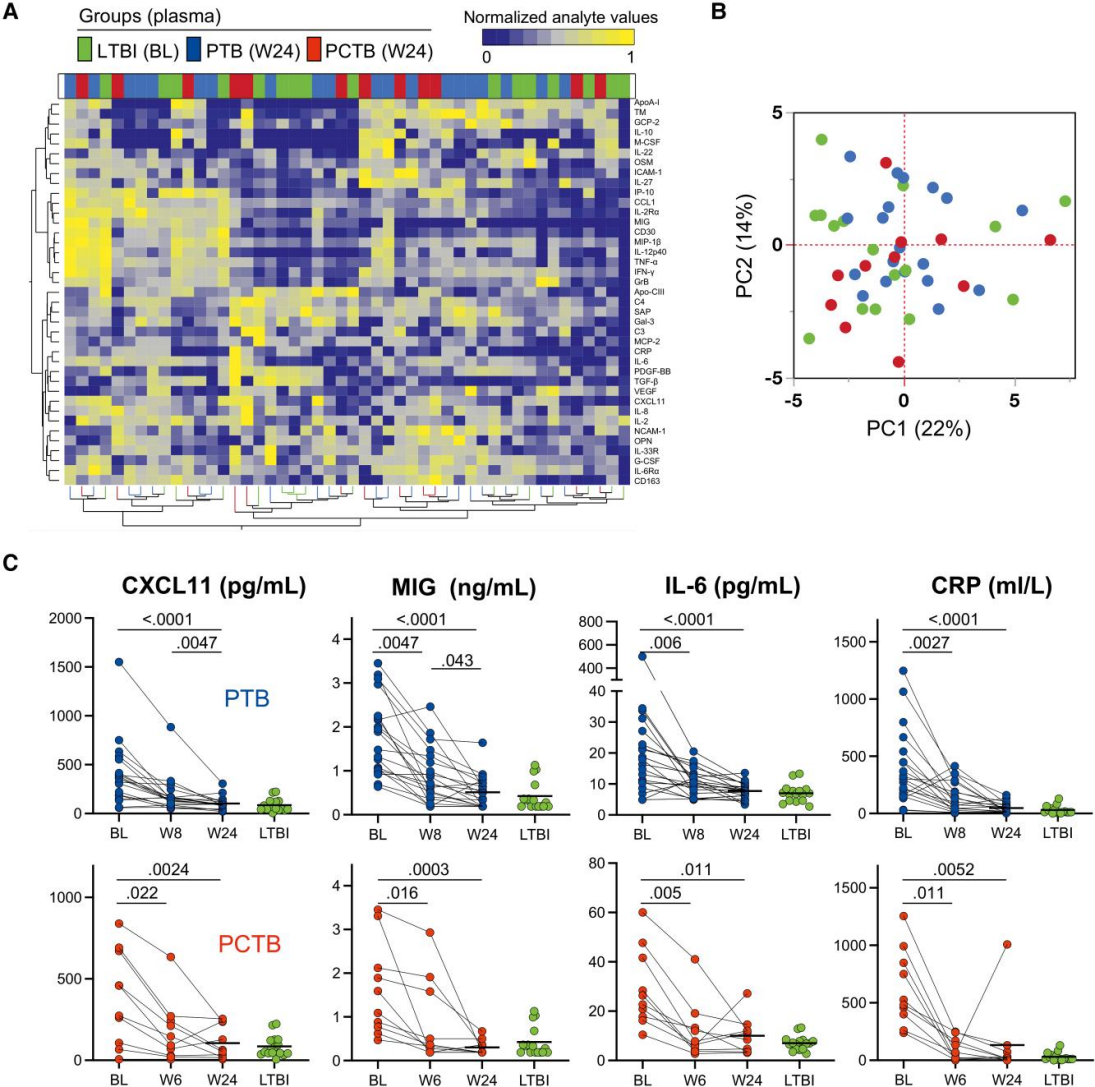
Analyte profiles in the different tuberculosis (TB) groups before, during, and after TB treatment. *A*, Nonsupervised 2-way hierarchical cluster analysis (Ward method) was employed to grade the TB groups using the 39 analytes (see Materials and Methods for expansions of abbreviations of analytes). TB status (pericardial TB [PCTB] in red, pulmonary TB [PTB] in blue, and latent TB infection [LTBI] in green) of each patient is indicated at the top of the dendrogram. Data are depicted as a heatmap colored from minimum to maximum normalized values detected for each marker. *B*, Principal component analysis on correlations based on the 39 analytes was used to explain the variance of the data distribution in the cohort. Each dot represents a participant. The 2 axes represent principal components 1 (PC1) and 2 (PC2). Their contribution to the total data variance is shown as a percentage. *C*, Representative graphs showing the change of concentrations of CXCL11, MIG, IL-6, and CRP with treatment and no statistical difference between week 24 (W24) post–treatment initiation and LTBI in both PTB and PCTB groups, respectively. Statistical comparisons were performed using a Friedman test, adjusted for multiple comparisons (Dunn test) for baseline (BL) vs week 6 (W6)/week 8 (W8), BL vs W24, and W6/W8 vs W24, and the Mann-Whitney test to compare LTBI with W24. *P* values were adjusted using the Benjamini-Hochberg multiple testing correction.

Monitoring of TB treatment response is challenging mainly due to the lack of specific and sensitive blood-based tools. In the current study, we examined the effect of TB treatment on the expression of inflammation markers. First, we compared the overall systemic inflammatory profile in participants with LTBI and in active TB patients (PTB and PCTB) 24 weeks after TB treatment initiation using unsupervised hierarchical clustering (Figure 5*A*) and PCA (Figure 5*B*). No specific clustering was observed between the groups, showing a global normalization of the inflammation signature at treatment completion. Furthermore, we performed univariate analysis comparing the level of expression of each analyte at baseline (before TB treatment initiation), week 6 or 8, and week 24 post–treatment initiation (Supplementary Figure 5 and Supplementary Table 3). Of the 39 measured analytes, 13 showed significant reduction between baseline, week 6/8, and/or week 24 in both the PTB and PCTB groups (Supplementary Figure 5*A* and Supplementary Table 3). An additional 8 analytes showed reduction between the 3 time points in the PTB group only (Supplementary Figure 5*B* and Supplementary Table 3).

Representative plots of analytes, including CXCL11, MIG, IL-6, and CRP, depict the significant reduction of expression of analytes with TB treatment from baseline, week 6/8 to end of treatment (week 24) in both PTB and PCTB groups (Figure 5*C*). These data suggest that the overall inflammatory profile normalized upon TB treatment completion in both PTB and PCTB.

### Comparison of HLA-DR Expression and Biosignatures Derived From Soluble Analytes in Discriminating LTBI From Active TB

Previous studies have shown the potential of blood-based markers to distinguish LTBI from active TB, including biosignatures derived from soluble markers and HLA-DR expression on *Mtb*-specific CD4 T cells [13, 14, 18, 19, 37]. While this study was not specifically designed to assess the performance of biosignature due to the limited number of participants included, we explored this aspect, wherein we assessed the ability of HLA-DR expression on *Mtb*-specific CD4 T cells to distinguish LTBI from PTB, PCTB, or any active TB (PTB + PCTB) and compared it with previously described biosignatures that included analytes measured in this study. We generated ROC curves from data obtained in *Mtb*-specific CD4 T cells. Consistent with previous reports, HLA-DR expression on *Mtb*-specific CD4 T cells showed a great capability to distinguish LTBI from PTB (*P* < .0001; area under the curve [AUC] = 0.97 [95% confidence interval {CI}, .92–1.00]; sensitivity: 97.75%, specificity: 100%, at an optimal cutoff of 48.5%) (Supplementary Figure 6*A* and 6*B*). Moreover, HLA-DR expression on *Mtb*-specific CD4 T cells also discriminated LTBI from PCTB (*P* < .0001; AUC = 0.94 [95% CI, .82–1.00]; sensitivity: 93.75%, specificity: 100%, at an optimal cutoff of 46.9%) and LTBI from any active TB (*P* < .0001; AUC = 0.96 [95% CI, .90–1.00]; sensitivity: 94.29%, specificity: 100%, at an optimal cutoff of 46.9%) (Supplementary Figure 6*A* and 6*B*).

We assessed the performance of previously described soluble biosignatures in our data set and compared the performance of these soluble biosignature performance to HLA-DR expression on *Mtb*-specific CD4 T cells. We identified 6 published biosignatures that include analytes measured in this study: IL-12p40 + IL-10 [19]; IFN-γ + IL-10 + IL-12p40 [19]; TNF-α + IL-12p40 [19]; CCL1 + CRP [13]; CCL1 + TNF-α [14]; and IL-6Rα + IL-2Rα [18].

These biosignatures discriminated LTBI from PTB with AUCs ranging from 0.72 to 0.98 and corresponding sensitivity and specificity ranging from 55% to 85% and 75% to 100%, respectively. They also discriminated LTBI from PCTB with AUCs ranging from 0.64 to 1.00 and corresponding sensitivity and specificity ranging from 61.11% to 83.33% and 62.5% to 93.75%, respectively, while they discriminated LTBI from any active TB (PTB + PCTB) with AUCs ranging from 0.69 to 0.98 and corresponding sensitivity and specificity ranging from 52.63% to 76.32% and 62.50% to 100%, respectively (Table 2). Detailed performances of these signatures in comparison to HLA-DR expression on *Mtb*-specific CD4 T cells are shown in Table 2.

**Table 2.** Comparing the Performance of HLA-DR Expression and Biosignatures From Literature in Discriminating Latent Tuberculosis (TB) Infection From TB Disease

Biosignature	AUC	Sensitivity, % (95% CI)	Specificity, % (95% CI)	Source
Performance of HLA-DR expression and biosignatures from literature in discriminating LTBI from PTB				
HLA-DR on *Mtb*-specific CD4 cells	0.97	94.74 (75.36–99.73)	100 (78.47–100)	Current study
CCL1 + CRP	0.98	65 (40.78–84.64)	100 (79.41–100)	Mutavhatsindi et al [13]
IL-6Rα + IL-2Rα	0.95	75 (50.90–91.34)	93.75 (69.77–99.84)	Eribo et al [18]
TNF-α + IL-12p40	0.92	85 (62.11–96.79)	93.75 (69.77–99.84)	Sutherland et al [19]
CCL1 + TNF-α	0.92	65 (40.78–84.61)	93.75 (69.77–99.84)	Chendi et al [14]
IFN-γ + IL-10 + IL-12p40	0.84	70 (45.72–88.11)	75 (46.62–92.73)	Sutherland et al [19]
IL-12p40 + IL-10	0.72	55 (31.53–76.94)	87.50 (61.65–98.45)	Sutherland et al [19]
Performance of HLA-DR expression and biosignatures from literature in discriminating LTBI from PCTB				
HLA-DR on *Mtb*-specific CD4 cells	0.94	93.75 (71.6–99.68)	100 (78.47–100)	Current study
CCL1 + CRP	1.00	83.33 (58.58–96.42)	100 (79.41–100)	Mutavhatsindi et al [13]
IL-6Rα + IL-2Rα	0.96	72.22 (46.52–90.31)	87.50 (61.65–98.45)	Eribo et al [18]
IFN-γ + IL-10 + IL-12p40	0.89	61.11 (35.75–82.70)	93.75 (69.77–99.84)	Sutherland et al [19]
CCL1 + TNF-α	0.85	61.11 (35.75–82.70)	93.75 (69.77–99.84)	Chendi et al [14]
TNF-α + IL-12p40	0.84	77.78 (52.36–93.59)	93.75 (69.77–99.84)	Sutherland et al [19]
IL-12p40 + IL-10	0.64	77.78 (52.36–93.59)	62.50 (35.43–84.80)	Sutherland et al [19]
Performance of HLA-DR and biosignatures from literature in discriminating LTBI from any active TB (PTB + PCTB)				
HLA-DR on *Mtb*-specific CD4 cells	0.96	94.29 (81.39–98.98)	100 (78.47–100)	Current study
CCL1 + CRP	0.98	71.05 (54.10–84.58)	100 (79.41–100)	Mutavhatsindi et al [13]
IL-6Rα + IL-2Rα	0.95	76.32 (59.76–88.56)	93.75 (69.77–99.84)	Eribo et al [18]
CCL1 + TNF-α	0.89	60.53 (43.39–75.96)	93.75 (69.77–99.84)	Chendi et al [14]
TNF-α + IL-12p40	0.88	76.32 (59.76–88.56)	93.75 (69.77–99.84)	Sutherland et al [19]
IFN-γ + IL-10 + IL-12p40	0.85	68.42 (51.35–82.50)	87.50 (61.65–98.45)	Sutherland et al [19]
IL-12p40 + IL-10	0.69	55.26 (38.30–71.38)	62.50 (35.43–84.80)	Sutherland et al [19]

Abbreviations: AUC, area under the curve; CCL, C-C motif ligand; CI, confidence interval; CRP, C-reactive protein; IFN-γ, interferon gamma; IL, interleukin; LTBI, latent tuberculosis infection; PCTB, pericardial tuberculosis; PTB, pulmonary tuberculosis; TNF-α, tumor necrosis factor alpha.

None of these biosignatures outperformed HLA-DR expression on *Mtb*-specific CD4 T cells in discriminating LTBI from the disease groups (Table 2). These findings suggest that HLA-DR is a better biomarker than soluble markers for discriminating between the different TB groups.

## DISCUSSION

Extrapulmonary TB represents a small but significant proportion of all TB cases globally, particularly in PWH, and is frequently difficult to diagnose. However, immune and inflammatory responses at the site of disease remain understudied. In this study, we compared the TB-associated inflammatory response in PWH between latent, pulmonary, and pericardial TB infection. We also compared the inflammatory signature in blood and at site of disease (ie, PCF) in PCTB patients. Moreover, we measured HLA-DR expression on *Mtb*-specific CD4 T cells from whole blood and compared its diagnostic potential to previously described biosignatures derived from different combinations of soluble markers.

We show that PTB in PWH is characterized by increased systemic inflammation compared to persons with LTBI. This is in accordance with previous reports showing elevated inflammatory markers (eg, CRP, IP-10, IFN-γ, CCL1, and VEGF) in unstimulated plasma or serum in active TB compared to LTBI or other respiratory diseases regardless of HIV-1 status [13, 14, 16]. In HIV-1–negative individuals, distinct inflammatory profiles in PTB versus EPTB have been reported, which were speculated to be the consequence of differences between disseminated versus more localized infection [40]. However, here, we observed a similar inflammatory profile in PTB individuals with HIV-1 and PCTB individuals with HIV-1. These differences may be explained by the different analytes measured in the Vinhaes et al [40] study and the current study, with only 7 analytes overlapping between the 2 studies (namely, IL-2, IL-6, IL-8, IL-10, IL-27, TNF-α, and IFN-γ). Moreover, the Vinhaes et al study included patients with different types of EPTB (including pleural TB, TB lymphadenitis, and miliary TB), whereas our study focused exclusively on PCTB patients.

To improve our understanding of immunological mechanisms at the disease site, we compared inflammatory profile at disease site and in plasma. A study by Matthews et al [20], assessing the inflammatory response at the disease site, showed compartmentalization of inflammatory proteins (including IL-6, IL-8, and IFN-γ) in PCF compared to blood. Our results are in accordance with this study, showing that inflammation was greater at the site of disease compared to the periphery and further demonstrate that there was a partial mirroring of the innate-associated inflammatory response (eg, CCL1, IL-12p40, TGF-β, and IL-8) between blood and disease site. Interestingly, Th1 cytokine levels (IFN-γ and IL-2) in PCF did not correlate with plasma levels. We previously reported that there was no correlation between the frequency of *Mtb*-specific CD4 T cells in blood and PCF [41], and recent data from murine model suggests that the rate of migration of T cells to the disease site is mostly regulated by the pattern of chemokine receptors they expressed [42].

TB diagnosis is challenging due to the lack of rapid, accurate, blood-based diagnostic tests. HLA-DR expression on *Mtb*-specific CD4 T cells has been shown to be a robust marker in discriminating latent TB from active TB [37–39] and EPTB [43]. In this study, we observed HLA-DR to be significantly highly expressed in blood of active TB compared to LTBI; it was also highly expressed at the site of disease (PCF) in PCTB participants compared to blood of the same participants. Our findings agree with those of previously published studies [37–39, 43] and further suggest that the extent of activation of infiltrating CD4 T cells is associated with the inflammatory profile at the disease site.

Several biosignatures consisting of host soluble inflammatory markers (measured in blood) have been described as promising tools for TB diagnosis [13, 14, 18, 19]. Here, we used our cohort as a validation cohort to compare their performance in discriminating LTBI from active TB, and several previously identified biosignatures continued to show promise in our cohort. However, none of these biosignatures showed better performance compared to the measure of HLA-DR expression on *Mtb*-specific CD4 T cells, which met the World Health Organization target product profile recommendations for a point-of-care, non-sputum-based triage test [44]. These data further emphasize the role of HLA-DR as a promising biomarker for TB diagnosis.

Sputum culture conversion at 2 months post–treatment initiation remains the most widely used tool for the evaluation of TB treatment response [45, 46]. However, in individuals with PCTB who are sputum smear or culture negative for *Mtb*, monitoring of treatment response is solely assessed clinically as there are no validated blood biomarkers to assist in this regard. Changes in blood biomarker levels during antitubercular treatment in either PTB or EPTB cases have been previously reported in a number of prospective studies [16, 47–50], showing the normalization of several inflammatory markers (such as CRP, IP-10, CCL1, IFN-γ, and TNF-α) after successful TB treatment. Our findings are in accordance with these results and add to current knowledge, showing that the concentrations of several of the biomarkers tested (21 of 39 and 13 of 39) decreased at treatment completion to levels observed in LTBI participants in both the PTB and PCTB groups, respectively. The discrepancy in the normalization of inflammatory profile after treatment between PTB and PCTB could be related to disease severity, where disseminated disease has been shown to present with elevated systemic bacterial burden and higher mortality [51] and limited drug penetration at the site of disease. Thus, our study confirms that measuring blood biomarkers may have utility to monitor treatment response in both PTB and EPTB.

Our study has several limitations. First, most of the participants were PWH; we were thus unable to define the impact of HIV-1 infection on TB-induced inflammatory profiles. Second, it is possible that other comorbidities (eg, diabetes or hypertension) may also influence patients’ inflammatory profile. Third, we did not have long-term follow-up clinical data to identify potential TB relapse, so long-term outcome could not be related to inflammatory profiles. Fourth, the current study was not designed to identify novel diagnostic markers; thus, we confined our analysis to previously described blood-based biomarkers. Further studies will be necessary to confirm the enhanced performance of HLA-DR over soluble biomarkers as a TB diagnostic tool. Fifth, as some LTBI participants may progress to active TB, it remains to be defined whether biomarkers derived from the measurements of soluble factors or phenotype of *Mtb*-specific cells would be relevant for the identification of people who are at risk for progression. Sixth, as the Mtb300 pool includes *Mtb*-derived peptides, which are also found in nontuberculous mycobacteria (NTM), NTM coinfection in active TB patients remains a possibility. Finally, further experiments including patients with nontuberculous pericardial effusion will be necessary to define whether the observed inflammatory signatures in plasma and at site of disease are TB specific.

Regardless of the limitations, our results show that in PWH with advanced immunosuppression, PCTB and PTB share similar inflammatory signature in plasma, and active TB disease disrupts HIV-1–associated systemic inflammation. This immunological interaction between HIV and *Mtb* could play a role in one disease accelerating the disease progression of the other. These results also reveal that the inflammatory profiles at the site of disease are distinct from peripheral blood, with elevated inflammation observed at the site of disease, though some markers strongly correlate between the 2 compartments. Furthermore, upon completion of TB treatment, levels of soluble analytes normalized; last, we showed that in PWH, assessing the expression of HLA-DR on *Mtb*-specific CD4 T cells had a better potential to discriminate PCTB and PTB from LTBI compared to biosignatures derived from soluble markers.

## Supplementary Material

ofad128_Supplementary_Data

## Supplementary Data

Supplementary materials are available at *Open Forum Infectious Diseases* online. Consisting of data provided by the authors to benefit the reader, the posted materials are not copyedited and are the sole responsibility of the authors, so questions or comments should be addressed to the corresponding author.

## Notes

***Author contributions*.** H. M.: Conceptualization, data curation, formal analysis, investigation, methodology, writing–original draft. E. D. B.: Conceptualization, data curation, writing–review and editing. S. R.: Methodology. P. H.: Data curation, writing–review and editing. M. C.: Data curation. A. S.: Conceptualization, funding acquisition. K. D. M.-B. and D. L. B.: Conceptualization, writing–review and editing. M. N. and R. J. W.: Conceptualization, funding acquisition, writing–review and editing. C. R.: Conceptualization, data curation, formal analysis, funding acquisition, investigation, methodology, writing–original draft.

***Acknowledgments***. The authors thank the study participants; the clinical staff at the Khayelitsha Site B Community Health Centre in Cape Town; and the laboratory staff at the Wellcome Centre for Infectious Disease Research in Africa at the University of Cape Town.

***Financial support.*** This work was supported by the European and Developing Countries Clinical Trials Partnership EDCTP2 program; the European Union's Horizon 2020 programme (Training and Mobility Action TMA2017SF-1951-TB-SPEC to C. R.); the National Institutes of Health (NIH) (R21AI148027 to C. R.); and the South African Medical Research Council (MRC-UFSP-1-IMPI-2 to M. N.). R. J. W. is supported by the Francis Crick Institute, which receives funds from Cancer Research UK (FC00110218), Wellcome (FC00110218) and the UK Medical Research Council (FC00110218). R. J. W. is also supported by Wellcome (203135) and the NIH (U01/115940, U01/152103). H. M. is supported by the National Research Foundation of South Africa (129614), CIDRI-Africa Fellowship, and in part by the Fogarty International Center, NIH (D43TW010559). D. L. B., K. D. M.-B., and A. S. are supported by the Division of Intramural Research, National Institute of Allergy and Infectious Diseases, NIH.

## References

[1] Joint United Nations Programme on HIV/AIDS . Global HIV and AIDS statistics—fact sheet. 2022. Available at: https://www.unaids.org/en/resources/fact-sheet. Accessed 31 January 2022.

[2] Sharma SK , Mohan A. Extrapulmonary tuberculosis. Indian J Med Res 2004; 120:316–53. 15520485

[3] Cagatay AA , Caliskan Y, Aksoz S, et al Extrapulmonary tuberculosis in immunocompetent adults. Scand J Infect Dis 2004; 36:799–806. 15764164 10.1080/00365540410025339

[4] Shafer RW , Kim DS, Weiss JP, Quale JM. Extrapulmonary tuberculosis in patients with human immunodeficiency virus infection. Medicine (Baltimore) 1991; 70:384–97. 1956280 10.1097/00005792-199111000-00004

[5] Rieder HL , Snider DE, Cauthen GM. Extrapulmonary tuberculosis in the United States. Am Rev Respir Dis 1990; 141:347–51. 2301852 10.1164/ajrccm/141.2.347

[6] Noubiap JJ , Agbor VN, Ndoadoumgue AL, et al Epidemiology of pericardial diseases in Africa: a systematic scoping review. Heart 2019; 105:180–8. 30415206 10.1136/heartjnl-2018-313922

[7] Reuter H , Burgess LJ, Doubell AF. Epidemiology of pericardial effusions at a large academic hospital in South Africa. Epidemiol Infect 2005; 133:393–9. 15962545 10.1017/s0950268804003577 PMC2870262

[8] Mayosi BM , Ntsekhe M, Bosch J, et al Prednisolone and *Mycobacterium indicus pranii* in tuberculous pericarditis. N Engl J Med 2014; 371:1121–30. 25178809 10.1056/NEJMoa1407380 PMC4912834

[9] Geldmacher C , Zumla A, Hoelscher M. Interaction between HIV and *Mycobacterium tuberculosis*: HIV-1-induced CD4 T-cell depletion and the development of active tuberculosis. Curr Opin HIV Aids 2012; 7:268–75. 22495739 10.1097/COH.0b013e3283524e32

[10] Riou C , Strickland N, Soares AP, et al HIV skews the lineage-defining transcriptional profile of *Mycobacterium tuberculosis*–specific CD4+ T cells. J Immunol 2016; 196:3006–18. 26927799 10.4049/jimmunol.1502094 PMC4799776

[11] Brenchley JM , Paiardini M, Knox KS, et al Differential Th17 CD4 T-cell depletion in pathogenic and nonpathogenic lentiviral infections. Blood 2008; 112:2826–35. 18664624 10.1182/blood-2008-05-159301 PMC2556618

[12] Clark S , Page E, Ford T, et al Reduced T(H)1/T(H)17 CD4 T-cell numbers are associated with impaired purified protein derivative-specific cytokine responses in patients with HIV-1 infection. J Allergy Clin Immunol 2011; 128:838–46.e5. 21745684 10.1016/j.jaci.2011.05.025

[13] Mutavhatsindi H , Van Der Spuy GD, Malherbe S, et al Validation and optimisation of host immunological bio-signatures for a point-of-care test for TB disease. Front Immunol 2021; 12:607827. 10.3389/fimmu.2021.607827 PMC7952865 33717089

[14] Chendi BH , Tveiten H, Snyders CI, et al CCL1 and IL-2Ra differentiate tuberculosis disease from latent infection irrespective of HIV infection in low TB burden countries. J Infect 2021; 83:433–43. 34333033 10.1016/j.jinf.2021.07.036

[15] Chegou NN , Sutherland JS, Malherbe S, et al Diagnostic performance of a seven-marker serum protein biosignature for the diagnosis of active TB disease in African primary healthcare clinic attendees with signs and symptoms suggestive of TB. Thorax 2016; 71:785–94. 27146200 10.1136/thoraxjnl-2015-207999

[16] Jacobs R , Malherbe S, Loxton AG, et al Identification of novel host biomarkers in plasma as candidates for the immunodiagnosis of tuberculosis disease and monitoring of tuberculosis treatment response. Oncotarget 2016; 7:57581–92. 27557501 10.18632/oncotarget.11420 PMC5295374

[17] Liu QY , Han F, Pan LP, Jia HY, Li Q, Zhang ZD. Inflammation responses in patients with pulmonary tuberculosis in an intensive care unit. Exp Ther Med 2018; 15:2719–26. 29456674 10.3892/etm.2018.5775 PMC5795479

[18] Eribo OA , Leqheka MS, Malherbe ST, et al Host urine immunological biomarkers as potential candidates for the diagnosis of tuberculosis. Int J Infect Dis 2020; 99:473–81. 32800854 10.1016/j.ijid.2020.08.019

[19] Sutherland JS , de Jong BC, Jeffries DJ, Adetifa IM, Ota MOC. Production of TNF-α, IL-12(p40) and IL-17 can discriminate between active TB disease and latent infection in a West African cohort. PLoS One 2010; 5:e12365. 10.1371/journal.pone.0012365 PMC2927558 20811496

[20] Matthews K , Deffur A, Ntsekhe M, et al A compartmentalized profibrotic immune response characterizes pericardial tuberculosis, irrespective of HIV-1 infection. Am J Respir Crit Care Med 2015; 192:1518–21. 26669475 10.1164/rccm.201504-0683LE PMC4731721

[21] Reuter H , Burgess LJ, Carstens ME, Doubell AF. Characterization of the immunological features of tuberculous pericardial effusions in HIV positive and HIV negative patients in contrast with non-tuberculous effusions. Tuberculosis 2006; 86:125–33. 16360340 10.1016/j.tube.2005.08.018

[22] Yang Q , Cai Y, Zhao W, et al IP-10 and MIG are compartmentalized at the site of disease during pleural and meningeal tuberculosis and are decreased after antituberculosis treatment. Clin Vaccine Immunol 2014; 21:1635–44. 25274803 10.1128/CVI.00499-14 PMC4248780

[23] Mayosi BM , Burgess LJ, Doubell AF. Tuberculous pericarditis. Circulation 2005; 112:3608–16. 16330703 10.1161/CIRCULATIONAHA.105.543066

[24] Hanekom WA , Hughes J, Mavinkurve M, et al Novel application of a whole blood intracellular cytokine detection assay to quantitate specific T-cell frequency in field studies. J Immunol Methods 2004; 291:185–95. 15345316 10.1016/j.jim.2004.06.010

[25] Arlehamn CSL , McKinney DM, Carpenter C, et al A quantitative analysis of complexity of human pathogen-specific CD4 T cell responses in healthy *M. tuberculosis* infected South Africans. PLoS Pathog 2016; 12:e1005760. 10.1371/journal.ppat.1005760 PMC4943605 27409590

[26] Robison HM , Chapman CA, Zhou H, et al Risk assessment of latent tuberculosis infection through a multiplexed cytokine biosensor assay and machine learning feature selection. Sci Rep 2021; 11:20544. 34654869 10.1038/s41598-021-99754-3 PMC8520014

[27] Mutavhatsindi H , Riou C. Protocol to quantify and phenotype SARS-CoV-2-specific T cell response using a rapid flow-cytometry-based whole blood assay. STAR Protoc 2022; 3:101771. 10.1016/j.xpro.2022.101771 PMC9510067 36272131

[28] Fluss R , Faraggi D, Reiser B. Estimation of the Youden index and its associated cutoff point. Biom J 2005; 47:458–72. 16161804 10.1002/bimj.200410135

[29] Benjamini Y , Hochberg Y. Controlling the false discovery rate: a practical and powerful approach to multiple testing. J R Stat Soc Ser B Methodol 1995; 57:289–300.

[30] Wang D , Tong X, Wang L, et al The association between osteopontin and tuberculosis: a systematic review and meta-analysis. PLoS One 2020; 15:e0242702. 10.1371/journal.pone.0242702 PMC7710079 33264357

[31] Muema DM , Akilimali NA, Ndumnego OC, et al Association between the cytokine storm, immune cell dynamics, and viral replicative capacity in hyperacute HIV infection. BMC Med 2020; 18:81. 32209092 10.1186/s12916-020-01529-6 PMC7093991

[32] Teigler JE , Leyre L, Chomont N, et al Distinct biomarker signatures in HIV acute infection associate with viral dynamics and reservoir size. JCI Insight 2018; 3:e98420. 10.1172/jci.insight.98420 PMC6018979 29769442

[33] Borges ÁH , O’Connor JL, Phillips AN, et al Factors associated with plasma IL-6 levels during HIV infection. J Infect Dis 2015; 212:585–95. 25722296 10.1093/infdis/jiv123 PMC4598808

[34] Roberts L , Passmore JAS, Williamson C, et al Plasma cytokine levels during acute HIV-1 infection predict HIV disease progression. AIDS 2010; 24:819–31. 20224308 10.1097/QAD.0b013e3283367836 PMC3001189

[35] Bunjun R , Soares AP, Thawer N, et al Dysregulation of the immune environment in the airways during HIV infection. Front Immunol 2021; 12:707355. 10.3389/fimmu.2021.707355 PMC8278481 34276702

[36] Rizzardi GP , Barcellini W, Tambussi G, et al Plasma levels of soluble CD30, tumour necrosis factor (TNF)-alpha and TNF receptors during primary HIV-1 infection: correlation with HIV-1 RNA and the clinical outcome. AIDS 1996; 10:F45–50. 8931778 10.1097/00002030-199611000-00001

[37] Adekambi T , Ibegbu CC, Cagle S, et al Biomarkers on patient T cells diagnose active tuberculosis and monitor treatment response. J Clin Invest 2015; 125:1827–38. 25822019 10.1172/JCI77990 PMC4598074

[38] Riou C , Berkowitz N, Goliath R, Burgers WA, Wilkinson RJ. Analysis of the phenotype of *Mycobacterium tuberculosis*–specific CD4^+^ T cells to discriminate latent from active tuberculosis in HIV-uninfected and HIV-infected individuals. Front Immunol 2017; 8:968. 28848561 10.3389/fimmu.2017.00968 PMC5554366

[39] Riou C , Du Bruyn E, Ruzive S, et al Disease extent and anti-tubercular treatment response correlates with *Mycobacterium tuberculosis*-specific CD4 T-cell phenotype regardless of HIV-1 status. Clin Transl Immunol 2020; 9:e1176. 10.1002/cti2.1176 PMC7520805 33005414

[40] Vinhaes CL , Oliveira-de-Souza D, Silveira-Mattos PS, et al Changes in inflammatory protein and lipid mediator profiles persist after antitubercular treatment of pulmonary and extrapulmonary tuberculosis: a prospective cohort study. Cytokine 2019; 123:154759. 10.1016/j.cyto.2019.154759 PMC6739167 31226436

[41] Du Bruyn E , Ruzive S, Howlett P, et al Profile of *Mycobacterium tuberculosis*–specific CD4 T cells at the site of disease and blood in pericardial tuberculosis. bioRxiv [Preprint]. Posted online 13 May 2022. doi:10.1101/2022.05.12.491749 PMC9692124 36439130

[42] Hoft SG , Sallin MA, Kauffman KD, Sakai S, Ganusov VV, Barber DL. The rate of CD4 T cell entry into the lungs during *Mycobacterium tuberculosis* infection is determined by partial and opposing effects of multiple chemokine receptors. Infect Immun 2019; 87:e00841-18. 10.1128/IAI.00841-18 PMC6529656 30962399

[43] Silveira-Mattos PS , Barreto-Duarte B, Vasconcelos B, et al Differential expression of activation markers by *Mycobacterium tuberculosis*–specific CD4^+^ T cell distinguishes extrapulmonary from pulmonary tuberculosis and latent infection. Clin Infect Dis 2020; 71:1905–11. 31665254 10.1093/cid/ciz1070 PMC8463092

[44] World Health Organization . High priority target product profiles for new tuberculosis diagnostics: report of a consensus meeting, 28–29 April 2014, Geneva, Switzerland. WHO/HTM/TB/2014.18. 2014. Available at: https://apps.who.int/iris/handle/10665/135617. Accessed 3 June 2022.

[45] Mitchison DA . Assessment of new sterilizing drugs for treating pulmonary tuberculosis by culture at 2 months. Am Rev Respir Dis 1993; 147:1062–3. 8466107 10.1164/ajrccm/147.4.1062

[46] Wallis RS , Pai M, Menzies D, et al Biomarkers and diagnostics for tuberculosis: progress, needs, and translation into practice. Lancet 2010; 375:1920–37. 20488517 10.1016/S0140-6736(10)60359-5

[47] Martins C , Gama ACC, Valcarenghi D, et al Markers of acute-phase response in the treatment of pulmonary tuberculosis. J Bras Patol Med Lab 2014; 50:428–33.

[48] Chendi BH , Snyders CI, Tonby K, et al A plasma 5-marker host biosignature identifies tuberculosis in high and low endemic countries. Front Immunol 2021; 12:608846. 10.3389/fimmu.2021.608846 PMC7958880 33732236

[49] Liang L , Shi R, Liu X, et al Interferon-gamma response to the treatment of active pulmonary and extra-pulmonary tuberculosis. Int J Tuberc Lung Dis 2017; 21:1145–9. 28911359 10.5588/ijtld.16.0880 PMC6310125

[50] Bai X-J , Li H-M, Yang Y-R, Zhang J-X, Liang Y, Wu X-Q. Cytokine and soluble adhesion molecule profiles and biomarkers for treatment monitoring in re-treated smear-positive patients with pulmonary tuberculosis. Cytokine 2018; 108:9–16. 29554572 10.1016/j.cyto.2018.03.009

[51] Schutz C , Barr D, Andrade BB, et al Clinical, microbiologic, and immunologic determinants of mortality in hospitalized patients with HIV-associated tuberculosis: a prospective cohort study. PLoS Med 2019; 16:e1002840. 10.1371/journal.pmed.1002840 PMC6611568 31276515

