# Supplementary material for: Blood and Site of Disease Inflammatory Profiles Differ in Patients With Pericardial Tuberculosis and Human Immunodeficiency Virus Type 1
Source: Open Forum Infect Dis. 2023 Mar 9;10(3):ofad128. doi: 10.1093/ofid/ofad128 (PMC10043131; doi:10.1093/ofid/ofad128)
Supplement: ofad128_Supplementary_Data [file ofad128_supplementary_data.zip › Supplementary tables_20230220.docx]

**Supplementary table 1.** **Baseline median levels and interquartile ranges (IQR) of analytes detected in participants with PCTB, PTB and LTBI.**

|  |  |  |  |  | Adjusted P-Values | | | |
| --- | --- | --- | --- | --- | --- | --- | --- | --- |
| **Analytes** | **LTBI**  **n=16** | **PTB**  **n=20** | **PCTB**  **n=18** | **PTB & PCTB**  **n=38** | LTBI vs PTB | LTBI vs PCTB | PTB vs PCTB | LTBI vs All aTB |
| **Inflammation regulation** | | | | | | | | |
| GrB^†^ | 14.30  (11.07-25.48) | 23.22  (19-31.58) | 24.98  (18.71-29.57) | 24.37  (18.99-29.57) | **0.0167** | **0.0483** | >0.99 | **0.0057** |
| IL-2^†^ | 6.38  (4.41-8.983) | 8.87  (6.473-12.76) | 9.625  (6.27-11.25) | 9.25  (6.27-12) | 0.1331 | 0.1241 | >0.99 | **0.0360** |
| IL-8^†^ | 4.855  (3.693-6.235) | 8.73  (4.595-10.25) | 9.095  (5.918-13.09) | 8.73  (5.188-10.86) | **0.0263** | **0.0040** | >0.99 | **0.0017** |
| IL-12p40 ^†^ | 91.66  (38.48-131.7) | 188.8  (90.71-282.0) | 113.7  (77.04-202.3) | 140.4  (77.04-242.9) | 0.0594 | >0.99 | 0.4508 | 0.0942 |
| M-CSF^†^ | 4.13  (4.13-92.75) | 32  (4.13-19 3.8) | 4.13  (4.13-103.7) | 4.13  (4.13-137.8) | >0.99 | >0.99 | 0.5674 | 0.8245 |
| TNF-⍺^†^ | 6.09  (5.158-8.313) | 12.38  (10.25-15.12) | 10.57  (8.570-12.88) | 11.44  (8.680-14.37) | **0.0002** | **0.0252** | 0.491 | **<0.0001** |
| TGF-β^§^ | 4.865  (3.788-20.51) | 5.9  (2.965-15.94) | 6.645  (3.330-23.21) | 6.195  (3.330-18.79) | >0.99 | >0.99 | >0.99 | 0.9510 |
| C3^‡^ | 121.9  (85.9-188.5) | 284.8  (188.6-499.9) | 225.4  (137.1-435.4) | 248.5  (180.8-454.6) | **0.0006** | **0.0468** | 0.5989 | **0.0009** |
| C4^‡^ | 339.8  (256.9-448.7) | 479.8  (365.4-588.4) | 385.2  (297.5-503.4) | 430.7  (342.2-544.6) | **0.0243** | >0.99 | 0.1963 | 0.0837 |
| CRP^‡^ | 12.11  (10.88-41.73) | 301  (167.4-518.2) | 631.1  (432.2-965.1) | 447.9  (235.0-810.6) | **0.0002** | **<0.0001** | 0.1408 | **<0.0001** |
| SAP^‡^ | 7.64  (5.48-10.95) | 11.48  (9.86-12.85) | 9.61  (7.25-11.43) | 10.46  (8.21-12.69) | **0.0253** | >0.99 | 0.2256 | 0.0695 |
| IL-22^§^ | 0.32  (0.21-0.67) | 0.24  (0.15-0.50) | 0.31  (0.17-0.53) | 0.27  (0.16-0.51) | >0.99 | >0.99 | >0.99 | 0.5832 |
| Gal-3^§^ | 5.99  (4.80-7.65) | 10.56  (8.84-12.76) | 9.44  (7.01-11.11) | 9.62  (8.01-12.45) | **<0.0001** | **0.0038** | 0.7957 | **<0.0001** |
| ICAM-1^§^ | 111.6  (47.92-281.0) | 235.5  (147.3-508.6) | 254.4  (141.7-492.3) | 246.5  (146.1-490.4) | 0.0548 | 0.0647 | >0.99 | **0.0144** |
| NCAM-1^§^ | 82.79  (66.01-132.0) | 87.06  (71.61-128.5) | 114.3  (77.44-143.1) | 99.97  (76.82-132.2) | >0.99 | >0.99 | 0.9726 | 0.7161 |
| G-CSF^§^ | 0.140  (0.12-0.18) | 0.18  (0.14-0.21) | 0.14  (0.1-0.15) | 0.15  (0.12-0.19) | 0.1429 | >0.99 | **0.0104** | 0.5832 |
| IFN-γ ^†^ | 27.81  (21.51-32.20) | 36.61  (31.82-49.34) | 38.03  (32.73-57.08) | 37.35  (32.73-51.38) | **0.0058** | **0.0018** | >0.99 | **0.0003** |
| IL-6^†^ | 6.885  (4.62-8.163) | 17.13  (10.49-26.05) | 27.36  (17.72-47.42) | 21.28  (14.35-34.89) | **0.0017** | **<0.0001** | 0.0980 | **<0.0001** |
| IL-10^†^ | 0.16  (0.16-0.7675) | 0.16  (0.16-1.07) | 0.16  (0.16-0.46) | 0.16  (0.16-0.89) | >0.99 | >0.99 | 0.8161 | 0.8234 |
| IL-27^†^ | 149.7  (109.4-211.5) | 406.1  (223.2-565.8) | 405.6  (270.7-719.6) | 406.1  (253.0-691.7) | **0.0001** | **0.0001** | >0.99 | **<0.0001** |
| VEGF^†^ | 15.86  (4.64-38) | 39.4  (25.48-106.9) | 55.05  (39.92-98.64) | 48.24  (25.71-101.2) | 0.0503 | **0.0063** | >0.99 | **0.0039** |
| **Chemokines** | | | | | | | | |
| MIG^§^ | 0.31  (0.19-0.6) | 1.69  (1.02-2.24) | 1.69  (0.85-2.92) | 1.69  (0.96-2.4) | **<0.0001** | **0.0002** | >0.99 | **<0.0001** |
| MCP-2^†^ | 58.77  (33.53-72.68) | 85.79  (61.34-117) | 75.46  (62.2-96.47) | 79.1  (62.2-102.6) | **0.0089** | 0.1739 | 0.8660 | **0.0098** |
| GCP-2^†^ | 177.8  (99.72-351.2) | 203.8  (144-335.6) | 171.8  (98.85-298.1) | 191.3  (121.1-312.9) | >0.99 | >0.99 | 0.8647 | 0.8866 |
| CXCL11^†^ | 58.34  (43.09-125.5) | 359.2  (186.4-519.7) | 365.9  (189.2-535.3) | 359.2  (187.5-526.6) | **0.0001** | **0.0002** | >0.99 | **<0.0001** |
| MIP-1β^†^ | 256.8  (191.9-305.6 | 357.9  (306.3-404.8) | 302.8  (251.2-375.8) | 324.4  (272.6-388.5) | **0.0061** | 0.4046 | 0.3271 | **0.0144** |
| CCL1^†^ | 3.50  (2.3-4.29) | 16.01  (8.01-42.45) | 13.69  (9.89-26.77) | 14.45  (9.89-33.48) | **<0.0001** | **<0.0001** | >0.99 | **<0.0001** |
| IP-10^†^ | 27.53  (19.74-98.70) | 224.9  (98.06-308.1) | 181.7  (87.29-353.9) | 224.5  (94.93-325.1) | **0.0002** | **0.0003** | >0.99 | **<0.0001** |
| **Fibrosis regulation** | | | | | | | | |
| OSM^†^ | 460.8  (319.5-537.5) | 521.1  (437.5-656) | 460.9  (391.5-546.5) | 491.2  (417.5-640.7) | 0.1445 | >0.99 | 0.1877 | 0.2939 |
| IL-33R^§^ | 6.48  (4.43-8.9) | 17.25  (11.78-28.89) | 39.25  (22.52-76.88) | 23.56  (14.48-59.6) | **0.0018** | **<0.0001** | 0.0957 | **0.0003** |
| OPN^§^ | 14.83  (12.46-23.52) | 25.9  (11.05-41.96) | 47.4  (23.11-77.78) | 31.49  (11.6-55.53) | 0.3743 | **0.0068** | 0.3009 | **0.0173** |
| PDGF-BB^§^ | 3.49  (1.93-4.97) | 5.23  (2.34-8.74) | 4.22  (2.92-6.78) | 4.69  (2.86-7.82) | 0.1829 | 0.5483 | >0.99 | 0.0999 |
| TM^§^ | 0.03  (0.02-0.098) | 0.05  (0.02-0.14) | 0.03  (0.02-0.08) | 0.04  (0.02-0.11) | 0.5632 | >0.99 | 0.6208 | 0.5364 |
| **Chemokine and protein receptors** | | | | | | | | |
| CD163^§^ | 614.5  (323.5-827.2) | 829.4  (428.5-1582) | 568.2  (382.2-915.4) | 691.9  (405.2-1132) | 0.3216 | >0.99 | 0.5708 | 0.3533 |
| IL-6R⍺^§^ | 29.51  (22.89-34.3) | 33.86  (28.81-38.72) | 28.10  (22.66-32.79) | 31.74  (25.53-37.25) | 0.0589 | >0.99 | **0.0319** | 0.2840 |
| CD30^†^ | 64.01  (35.13-92.94) | 145.3  (110.4-268.5) | 97.57  (75.11-154.3) | 133.7  (85.46-186.2) | **0.0011** | 0.2352 | 0.2100 | **0.0041** |
| IL-2R⍺^§^ | 0.33  (0.24-0.66) | 1.82  (1.13-2.4) | 1.47  (1.165-2.34) | 1.75  (1.165-2.36) | **<0.0001** | **<0.0001** | >0.99 | **<0.0001** |
| **Apolipoproteins** | | | | | | | | |
| ApoA-I^‡^ | 2050  (364.2-7118) | 5264  (175-14620) | 228.2  (104.1-23813) | 263.4  (153.2-15722) | >0.99 | >0.99 | >0.99 | 0.9510 |
| Apo-CIII^‡^ | 185.5  (64.7-284.3) | 177.9  (126.7-234.4) | 148.7  (75.01-359.8) | 163.6  (89.71-241.8) | >0.99 | >0.99 | >0.99 | 0.9510 |

Statistical comparisons were performed using a Kruskal-Wallis test adjusted for multiple comparisons (Dunn’s test) for LTBI vs PTB, LTBI vs PCTB and PTB vs PCTB and the Mann-Whitney test to compare LTBI and aTB (PCTB & PTB) with p-values adjusted using the Benjamini Hochberg multiple testing correction.

^†^: Values are shown in pg/ml, ^‡^: Values are shown in mg/L ^§^: Values are shown in ng/ml.

Abbreviations. LTBI: Latent tuberculosis infection, PTB: Pulmonary Tuberculosis, PCTB, Pericardial tuberculosis, aTB: Active tuberculosis, Apo: Apolipoprotein, C3: Complement component 3, C4: Complement component 4, CRP: C reactive protein, SAP: Serum amyloid protein, IL: Interleukin, PDGF: Platelet-derived growth factor, MCP: Monocyte chemotactic protein, ICAM: Intercellular adhesion molecule, NCAM: Neural cell adhesion molecule, IP-10: Interferon γ-induced protein 10 kDa, OPN: Osteopontin, CD: Cluster of differentiation, MIG: Monokine induced by gamma interferon, G-CSF: Granulocyte colony-stimulating factor, IFN: Interferon, OSM: Oncostatin M, VEGF: Vascular endothelial growth factor, MIP: Macrophage inflammatory protein, GCP-2: granulocyte chemotactic protein 2, CXCL11: C-X-C motif chemokine 11, CCL1: C-C Motif Chemokine Ligand 1, M-CSF: Macrophage colony-stimulating factor, TM: Thrombomodulin, TNF-⍺: Tumour necrosis factor alpha and TGF-β: Transforming growth factor beta.

**Supplementary table 2. Baseline median levels and interquartile ranges (IQR) of analytes detected in Plasma and PCF of participants with PCTB.**

| **Analytes** | **Plasma (n=18)** | **PCF (n=18)** | **Adjusted P-values** |
| --- | --- | --- | --- |
| **Inflammation regulation** | | | |
| GrB (pg/mL) | 24.98 (18.71-29.57) | 168.7 (120.8-311.5) | **<0.0001** |
| IL-2 (pg/mL) | 9.63 (6.27-11.25) | 37.05 (25.23-74.25) | **<0.0001** |
| IL-8 (pg/mL) | 9.10 (5.92-13.09) | 2163 (778.1-5155) | **<0.0001** |
| IL-12p40 (pg/mL) | 113.7 (77.04-202.3) | 613.0 (356.9-691.1) | **<0.0001** |
| M-CSF (pg/mL) | 4.13 (4.13-103.7) | 214.8 (4.130-1351) | **0.0033** |
| TNF-⍺ (pg/mL) | 10.57 (8.57-12.88) | 50.76 (22.9-100.6) | **<0.0001** |
| TGF-β (ng/mL) | 6.65 (3.33-23.21) | 11.7 (5.89-15.69) | 0.7337 |
| C3 (mg/L) | 225.4 (137.1-435.4) | 17.29 (6.143-30.77) | **<0.0001** |
| C4 (mg/L) | 385.2 (297.5-503.4) | 144.5 (109.9-220.4) | **<0.0001** |
| CRP (mg/L) | 631.1 (432.2-965.1) | 301.8 (106.5-484.6) | **<0.0001** |
| SAP (mg/L) | 9.60 (7.25-11.43) | 6.97 (5.21-8.59) | **0.0002** |
| IL-22 (ng/mL) | 0.31 (0.17-0.53) | 0.04 (0-0.19) | **<0.0001** |
| Gal-3 (ng/mL) | 9.44 (7.01-11.11) | 14.37 (12.71-29.31) | **<0.0001** |
| ICAM-1 (ng/mL) | 254.4 (141.7-492.3) | 269.7 (165.4-558.4) | **0.0455** |
| NCAM-1 (ng/mL) | 114.3 (77.44-143.1) | 87.11 (62.06-127.2) | **0.0417** |
| G-CSF (ng/mL) | 0.14 (0.1-0.15) | 0.66 (0.29-1.26) | **<0.0001** |
| IFN- γ (pg/mL) | 38.03 (32.73-57.08) | 1336 (455.8-2452) | **<0.0001** |
| IL-6 (pg/mL) | 27.36 (17.72-47.42) | 15571 (12341-19607) | **<0.0001** |
| IL-10 (pg/mL) | 0.16 (0.16-0.46) | 0.16 (0.16-3.715) | **0.0105** |
| IL-27 (pg/mL) | 405.6 (270.7-719.6) | 652.6 (477.8-835.3) | **0.0237** |
| VEGF (pg/mL) | 55.05 (39.92-98.64) | 1080 (747.6-1695) | **<0.0001** |
| **Chemokines** | | | |
| MIG (ng/mL) | 1.69 (0.85-2.9) | 76.20 (25.66-1009) | **<0.0001** |
| MCP-2 (pg/mL) | 75.46 (62.2-96.47) | 1493 (782.5-3514) | **<0.0001** |
| GCP-2 (pg/mL) | 171.8 (98.85-298.1) | 789.8 (283-2470) | **0.0002** |
| CXCL11 (pg/mL) | 365.9 (189.2-535.3) | 329.6 (177.4-1454) | 0.2479 |
| MIP-1β (pg/mL) | 302.8 (251.2-375.8) | 573.5 (492.4-638.9) | **<0.0001** |
| CCL1 (pg/mL) | 13.69 (9.89-26.77) | 313.8 (77.73-490.9) | **<0.0001** |
| IP-10 (pg/mL) | 181.7 (87.29-353.9) | 2131 (1867-2360) | **<0.0001** |
| **Fibrosis** **regulation** | | | |
| OSM (pg/mL) | 460.9 (391.5-546.5) | 1674 (1010-2943) | **<0.0001** |
| IL-33R (ng/mL) | 39.25 (22.52-76.88) | 24.52 (16.04-36.78) | **0.0298** |
| OPN (ng/mL) | 47.40 (23.11-77.78) | 53.75 (20.01-255.4) | 0.0907 |
| PDGF-BB (ng/mL) | 4.215 (2.920-6.778) | 0.08 (0.0475-0.1125) | **<0.0001** |
| TM (ng/mL) | 0.03 (0.0175-0.08) | 0.07 (0.0375-0.14) | **0.0486** |
| **Chemokine and protein receptors** | | | |
| CD163 (ng/mL) | 568.2 (382.2-915.4) | 1462 (1033-2072) | **<0.0001** |
| IL-6R⍺ (ng/mL) | 28.1 (22.66-32.79) | 7.59 (6.058-10.32) | **<0.0001** |
| CD30 (pg/mL) | 97.57 (75.11-154.3) | 309.4 (250.2-434.4) | **<0.0001** |
| IL-2R⍺ (ng/mL) | 1.47 (1.17-2.34) | 6.94 (4.94-8.70) | **<0.0001** |
| **Apolipoproteins** | | | |
| ApoA-I (mg/L) | 228.2 (104.1-23813) | 137.3 (78.28-17790) | 0.7204 |
| Apo-CIII (mg/L) | 148.7 (75.01-359.8) | 34.87 (11.8-597.5) | 0.6424 |

Statistical comparisons were performed using a Wilcoxon test and p-values were adjusted using the Benjamini Hochberg multiple testing correction.

Abbreviations. Apo: Apolipoprotein, C3: Complement component 3, C4: Complement component 4, CRP: C reactive protein, SAP: Serum amyloid protein, IL: Interleukin, TM: Thrombomodulin, PDGF: Platelet-derived growth factor, MCP: monocyte chemotactic protein, ICAM: Intercellular adhesion molecule, NCAM: Neural cell adhesion molecule, IP-10: Interferon γ-induced protein 10 kDa, OPN: Osteopontin, CD: Cluster of differentiation, MIG: Monokine induced by interferon gamma, G-CSF: Granulocyte colony-stimulating factor, IFN-γ: Interferon-gamma, OSM: Oncostatin M, VEGF: Vascular endothelial growth factor, MIP: Macrophage inflammatory protein, GCP-2: Granulocyte chemotactic protein 2, CXCL: C-X-C motif ligand, CCL: C-C motif ligand, M-CSF: Macrophage colony-stimulating factor, TNF-⍺: Tumour necrosis factor alpha, and TGF-β: Transforming growth factor beta.

**Supplementary table 3. Longitudinal median levels and interquartile ranges (IQR) of analytes detected in participants with PCTB and PTB at Baseline, Week 6/8 post treatment initiation and at the end of treatment (Week 24).**

|  | LTBI | PTB | | | P-Values | | | | PCTB | | | P-Values | | | |
| --- | --- | --- | --- | --- | --- | --- | --- | --- | --- | --- | --- | --- | --- | --- | --- |
| Analytes | **Baseline**  **n=16** | **Baseline**  **n=20** | **Week 8**  **n=20** | **Week 24**  **n=20** | BL vs W8 | BL vs W24 | W8 vs W24 | LTBI vs W24 | **Baseline**  **n=10** | **Week 6**  **n=10** | **Week 24**  **n=10** | BL vs W6 | BL vs W24 | W6 vs W24 | LTBI vs W24 |
| GrB^†^ | 14.30  (11.07-25.48) | 23.22  (19.0-31.58) | 19.26  (15.99-25.55) | 17.37  (14.71-20.09) | 0.207 | **0.002** | 0.342 | 0.5489 | 23.51  (16.27-29.57) | 16.19  (12.59-25.37) | 13.78  (10.69-17.3) | **0.030** | **0.0016** | >0.99 | 0.975 |
| IL-2^†^ | 6.38  (4.41-8.98) | 8.87  (6.47-12.76) | 7.8  (5.55-9.81) | 5.73  (3.59-7.74) | >0.99 | **0.001** | **0.027** | 0.6425 | 10.02  (7.94-12.25) | 7.04  (4.84-12.25) | 7.16  (5.54-9.25) | 0.1325 | 0.1325 | >0.99 | 0.990 |
| IL-8^†^ | 4.86  (3.70-6.24) | 8.73  (4.59-10.25) | 5.91  (3.49-9.99) | 5.21  (3.39-6.13) | 0.291 | **0.0002** | 0.0531 | 0.9146 | 7.89  (4.01-16.76) | 5.65  (3.78-15.51) | 6.27  (3.33-9.43) | >0.99 | 0.0566 | 0.2806 | 0.975 |
| IL-12p40 ^†^ | 91.66  (38.48-131.7) | 188.8  (90.71-282.0) | 131.7  (38.48-249.1) | 113.7  (46.28-174.5) | >0.99 | 0.1733 | 0.4642 | 0.7276 | 95.35  (77.04-202.3) | 100.7  (38.48-268.5) | 69.66  (61.87-136.1) | >0.99 | 0.6563 | 0.7907 | 0.990 |
| M-CSF^†^ | 4.13  (4.13-92.75) | 32.0  (4.13-193.8) | 4.13  (4.13-66.32) | 4.13  (4.13-74.99) | 0.707 | >0.99 | >0.99 | 0.9481 | 4.13  (4.13-103.7) | 45.04  (4.13-147.0) | 15.42  (4.13-92.63) | >0.99 | >0.99 | >0.99 | 0.990 |
| TNF-α^†^ | 6.09  (5.16-8.31) | 12.38  (10.25-15.12) | 9.96  (7.76-14.43) | 7.81  (7.12-9.53) | 0.8051 | **0.0008** | **0.0342** | 0.1577 | 10.68  (8.57-13.44) | 10.46  (5.33-14.79) | 6.61  (4.76-8.19) | 0.5391 | **0.0024** | 0.1325 | 0.990 |
| TGF-β^§^ | 4.87  (3.79-20.51) | 5.9  (2.96-15.94) | 5.18  (3.30-12.05) | 4.47  (2.71-8.95) | >0.99 | >0.99 | >0.99 | 0.5489 | 16.13  (3.73-35.59) | 11.55  (3.57-18.34) | 4.24  (3.80-9.04) | >0.99 | >0.99 | >0.99 | 0.990 |
| C3^‡^ | 121.9  (85.9-188.5) | 284.8  (188.6-499.9) | 306.6  (154.3-496.9) | 245.1  (154.3-306.1) | >0.99 | 0.6177 | 0.3415 | 0.0874 | 202.2  (119.7-323.3) | 242.7  (139.1-666.3) | 113.8  (67.98-228.8) | >0.99 | 0.2209 | 0.0760 | 0.990 |
| C4^‡^ | 339.8  (256.9-448.7) | 479.8  (365.4-588.4) | 395.7  (284.1-540.9) | 368.0  (296.7-443.8) | 0.1195 | **0.0047** | 0.8051 | 0.7669 | 407.3  (261.7-544.6) | 349.9  (268.7-429.5) | 277.3  (252.2-456.5) | >0.99 | >0.99 | >0.99 | 0.990 |
| CRP^‡^ | 12.11  (10.88-41.73) | 301.0  (167.4-518.2) | 87.15  (29.93-252.5) | 21.47  (12.18-74.6) | **0.0027** | **<0.0001** | 0.1733 | 0.4473 | 503.7  (365.1-884.6) | 63.70  (13.64-188.4) | 20.77  (11.14-87.52) | **0.011** | **0.0052** | >0.99 | 0.975 |
| SAP^‡^ | 7.64  (5.48-10.95) | 11.48  (9.86-12.85) | 9.57  (7.92-10.46) | 8.53  (7.3-9.39) | 0.2460 | **0.0342** | >0.99 | 0.7070 | 9.08  (5.85-12.71) | 9.73  (5.62-11.58) | 8.08  (6.06-8.89) | >0.99 | 0.7907 | >0.99 | 0.990 |
| IL-22^§^ | 0.32  (0.21-0.67) | 0.24  (0.15-0.50) | 0.32  (0.13-0.42) | 0.25  (0.143-0.59) | >0.99 | 0.7070 | >0.99 | 0.7070 | 0.25  (0.095-0.82) | 0.47  (0.35-1.2) | 0.32  (0.22-0.55) | **0.0156** | >0.99 | **0.0304** | 0.990 |
| Gal-3^§^ | 5.99  (4.80-7.65) | 10.56  (8.84-12.76) | 8.04  (7.02-9.68) | 6.23  (5.52-8.06) | **0.0133** | **<0.0001** | 0.0531 | 0.6271 | 8.52  (5.18-10.77) | 7.11  (3.79-9.010) | 4.0  (3.22-5.69) | 0.1720 | **0.0016** | 0.3526 | 0.753 |
| ICAM-1^§^ | 111.6  (47.92-281.0) | 235.5  (147.3-508.6) | 257.7  (157.2-526.6) | 264.7  (157.5-427.0) | >0.99 | >0.99 | 0.3415 | 0.0969 | 274.6  (150.3-527.5) | 207.3  (158.1-876.9) | 260.0  (143.7-700.0) | >0.99 | >0.99 | >0.99 | 0.753 |
| NCAM-1^§^ | 82.79  (66.01-132.0) | 87.06  (71.61-128.5) | 101.9  (79.87-165.8) | 117.4  (72.51-178.9) | 0.4642 | 0.0531 | >0.99 | 0.5489 | 107.0  (76.70-154.6) | 122.4  (90.52-168.4) | 141.7  (105.5-182.7) | 0.2209 | **0.0110** | 0.7907 | 0.753 |
| G-CSF^§^ | 0.14  (0.12-0.18) | 0.18  (0.14-0.21) | 0.19  (0.14-0.2) | 0.17  (0.14-0.23) | >0.99 | >0.99 | >0.99 | 0.4473 | 0.12  (0.10-0.15) | 0.17  (0.16-0.20) | 0.16  (0.13-0.22) | 0.2209 | 0.3526 | >0.99 | 0.975 |
| IFN-γ^†^ | 27.81  (21.51-32.2) | 36.61  (31.82-49.34) | 33.07  (27.51-41.32) | 29.39  (27.51-33.50) | 0.6177 | 0.0342 | 0.6177 | 0.5489 | 36.56  (31.12-59.33) | 33.08  (23.62-44.81) | 26.38  (24.89-31.99) | 0.0566 | **0.0076** | >0.99 | 0.990 |
| IL-6^†^ | 6.89  (4.62-8.16) | 17.13  (10.49-26.05) | 10.27  (7.27-13.26) | 7.95  (6.22-9.55) | **0.0061** | **<0.0001** | 0.4642 | 0.5489 | 24.54  (17.40-43.17) | 7.09  (4.14-14.68) | 9.89  (3.53-12.66) | **0.0052** | **0.0110** | >0.99 | 0.975 |
| IL-10^†^ | 0.16  (0.16-0.77) | 0.16  (0.16-1.07) | 0.40  (0.16-1.37) | 0.16  (0.16-0.64) | >0.99 | >0.99 | >0.99 | 0.7070 | 0.16  (0.16-0.46) | 0.64  (0.16-1.01) | 0.64  (0.16-1.13) | 0.2806 | 0.1325 | >0.99 | 0.975 |
| IL-27^†^ | 149.7  (109.4-211.5) | 406.1  (223.2-565.8) | 400.6  (210.2-589.5) | 371.9  (170.9-475.6) | 0.1733 | 0.1733 | >0.99 | **0.0273** | 405.6  (286.8-586.1) | 338.3  (198.6-475.3) | 214.5  (133.1-350.4) | >0.99 | 0.1325 | 0.5391 | 0.894 |
| VEGF^†^ | 15.86  (4.64-38.00) | 39.40  (25.48-106.9) | 22.68  (12.28-80.40) | 17.72  (9.05-24.71) | 0.1195 | **0.0008** | 0.3415 | 0.9481 | 59.11  (44.36-104.4) | 31.73  (13.69-76.9) | 19.30  (14.50-35.87) | 0.5391 | 0.1325 | >0.99 | 0.975 |
| MIG^§^ | 0.305  (0.19-0.60) | 1.685  (1.018-2.238) | 0.695  (0.37-1.303) | 0.43  (0.19-0.72) | **0.0047** | **<0.0001** | **0.0428** | 0.7070 | 1.34  (0.732-2.42) | 0.345  (0.19-1.663) | 0.19  (0.19-0.395) | **0.0156** | **0.0003** | 0.7907 | 0.975 |
| MCP-2^†^ | 58.77  (33.53-72.68) | 85.79  (61.34-117.0) | 67.32  (52.85-94.16) | 56.87  (35.20-71.04) | **0.0342** | **0.0001** | 0.3415 | 0.9686 | 78.67  (61.37-87.98) | 72.34  (41.11-79.14) | 42.22  (25.38-71.86) | >0.99 | 0.7907 | >0.99 | 0.975 |
| GCP-2^†^ | 177.8  (99.72-351.2) | 203.8  (144.0-335.6) | 173.7  (125.6-411.9) | 212.0  (135.5-296.1) | >0.99 | >0.99 | >0.99 | 0.7070 | 171.8  (117.1-221.2) | 271.0  (134.0-407.3) | 223.1  (116.5-314.4) | 0.2209 | 0.3526 | >0.99 | 0.990 |
| CXCL11^†^ | 58.34  (43.09-125.5) | 359.2  (186.4-519.7) | 143.0  (90.89-168.3) | 92.39  (83.09-119.8) | 0.0806 | **<0.0001** | **0.0047** | 0.5489 | 365.9  (96.34-676.7) | 118.7  (26.66-248.8) | 58.46  (17.84-237.7) | **0.0219** | **0.0024** | >0.99 | 0.990 |
| MIP-1β^†^ | 256.8  (191.9-305.6) | 357.9  (306.3-404.8) | 308.4  (279.6-403.3) | 276.6  (231.3-339.2) | 0.6177 | **0.0170** | 0.3992 | 0.5489 | 308.9  (228.9-375.8) | 284.8  (199.7-396.1) | 256.8  (194.1-279.1) | 0.7907 | 0.3526 | >0.99 | 0.990 |
| CCL1^†^ | 3.50  (2.30-4.288) | 16.01  (8.01-42.45) | 8.40  (3.99-18.46) | 4.49  (3.68-7.140 | **0.0342** | **<0.0001** | **0.0080** | 0.1577 | 13.90  (10.16-18.87) | 8.38  (3.84-29.22) | 3.92  (1.8-5.29) | >0.99 | **0.0010** | **0.0052** | 0.990 |
| IP-10^†^ | 27.53  (19.74-98.70) | 224.9  (98.06-308.1) | 92.13  (44.46-200.0) | 48.03  (28.65-97.27) | **0.008** | **<0.0001** | **0.0342** | 0.4473 | 130.5  (83.74-315.9) | 85.18  (38.12-143.9) | 43.13  (10.83-109.5) | 0.5391 | **0.0219** | 0.5391 | 0.990 |
| OSM^†^ | 460.8  (319.5-537.5) | 521.1  (437.5-656.0) | 521.1  (399.3-880.0) | 491.0  (344.6-686.6) | >0.99 | 0.7070 | 0.1733 | 0.6349 | 460.9  (391.5-720.9) | 497.6  (356.2-584.6) | 491.5  (273.0-568.5) | 0.7907 | 0.3526 | >0.99 | 0.990 |
| IL-33R^§^ | 6.48  (4.43-8.9) | 17.25  (11.78-28.89) | 12.84  (9.12-20.89) | 12.58  (6.32-14.25) | **0.0027** | **0.0027** | >0.99 | 0.0874 | 33.78  (22.52-70.52) | 12.03  (9.10-20.54) | 9.58  (6.47-12.16) | 0.2209 | **0.0110** | 0.7907 | 0.878 |
| OPN^§^ | 14.83  (12.46-23.52) | 25.90  (11.05-41.96) | 20.30  (9.895-39.58) | 23.00  (8.563-35.00) | 0.2460 | >0.99 | 0.3415 | 0.5489 | 47.40  (23.11-79.40) | 23.51  (10.88-40.52) | 22.68  (14.22-42.77) | 0.0760 | 0.4383 | >0.99 | 0.872 |
| PDGF-BB^§^ | 3.49  (1.93-4.97) | 5.23  (2.34-8.74) | 3.05  (1.83-7.38) | 2.29  (1.45-4.67) | 0.3415 | **0.0047** | 0.3415 | 0.6425 | 4.94  (2.64-8.13) | 3.25  (2.64-7.00) | 2.13  (0.97-3.78) | >0.99 | **0.0417** | 0.3526 | 0.975 |
| TM^§^ | 0.03  (0.02-0.10) | 0.05  (0.02-0.14) | 0.09  (0.02-0.15) | 0.10  (0.04-0.12) | >0.99 | >0.99 | >0.99 | 0.1293 | 0.03  (0.02-0.07) | 0.06  (0.03-0.13) | 0.105  (0.02-0.15) | 0.1009 | 0.0760 | >0.99 | 0.753 |
| CD163^§^ | 614.5  (323.5-827.2) | 829.4  (428.5-1582) | 792.8  (362.8-1225) | 823.2  (393.7-1077) | >0.99 | 0.2460 | 0.8051 | 0.4473 | 547.1  (366.3-1053) | 660.0  (343.2-1153) | 501.5  (267.0-1061) | >0.99 | 0.7907 | 0.3526 | 0.990 |
| IL-6Rα^§^ | 29.51  (22.89-34.30) | 33.86  (28.81-38.72) | 32.42  (27.77-38.98) | 32.36  (31.06-37.61) | >0.99 | >0.99 | 0.8051 | 0.0874 | 26.45  (22.66-33.61) | 30.34  (26.10-37.54) | 31.99  (26.35-35.77) | >0.99 | 0.5391 | >0.99 | 0.975 |
| CD30^†^ | 64.01  (35.13-92.94) | 145.3  (110.4-268.5) | 107.5  (73.42-256.2) | 104.7  (53.07-145.9) | >0.99 | **0.0133** | 0.1733 | 0.5489 | 94.36  (64.56-140.8) | 60.01  (42.69-156.3) | 51.40  (35.32-72.80) | >0.99 | 0.1325 | 0.5391 | 0.975 |
| IL-2Rα^§^ | 0.33  (0.24-0.66) | 1.82  (1.13-2.40) | 0.82  (0.54-1.47) | 0.68  (0.42-0.90) | **0.0342** | **<0.0001** | **0.0342** | 0.0874 | 1.43  (1.17-2.11) | 0.53  (0.365-1.12) | 0.49  (0.26-0.74) | 0.0760 | **0.0004** | 0.3526 | 0.975 |
| ApoA-I^‡^ | 2050  (364.2-7118) | 5264  (175-14620) | 5098  (335.2-7264) | 5519  (454.2-7081) | >0.99 | >0.99 | >0.99 | 0.6464 | 204.3  (101-29955) | 7496  (461-11029) | 6120  (249.1-8976) | >0.99 | >0.99 | >0.99 | 0.975 |
| Apo CIII^‡^ | 185.5  (64.70-284.3) | 177.9  (126.7-234.4) | 146.5  (86.05-198.0) | 125.2  (102.6-172.5) | >0.99 | 0.4642 | >0.99 | 0.5489 | 114.6  (78.89-376.5) | 188.8  (108.3-323.1) | 167.1  (96.66-250.8) | 0.7907 | >0.99 | >0.99 | 0.990 |

Week 24 was further compared and LTBI. Statistical comparisons were performed using a Friedman test adjusted for multiple comparisons (Dunn’s test) for BL v W6/8, BL v W24 and W6/8 v W24 for both PCTB and PTB groups and the Mann-Whitney test to compare LTBI with W24 in both PCTB and PTB with p-values adjusted using the Benjamini Hochberg multiple testing correction.

^‡^: Values are shown in mg/L, ^†^: Values are shown in pg/mL, ^§^: Values are shown in ng/mL.

Abbreviations. Apo: Apolipoprotein, C3: Complement component 3, C4: Complement component 4, CRP: C reactive protein, SAP: Serum amyloid protein, IL: Interleukin, TM: Thrombomodulin, PDGF: Platelet-derived growth factor, MCP: monocyte chemotactic protein, ICAM: Intercellular adhesion molecule, NCAM: Neural cell adhesion molecule, IP-10: Interferon γ-induced protein 10 kDa, OPN: Osteopontin, CD: Cluster of differentiation, MIG: Monokine induced by interferon gamma, G-CSF: Granulocyte colony-stimulating factor, IFN-γ: Interferon-gamma, OSM: Oncostatin M, VEGF: Vascular endothelial growth factor, MIP: Macrophage inflammatory protein, GCP-2: Granulocyte chemotactic protein 2, CXCL: C-X-C motif ligand, CCL: C-C motif ligand, M-CSF: Macrophage colony-stimulating factor, TNF-⍺: Tumour necrosis factor alpha, and TGF-β: Transforming growth factor beta.
