# Supplementary material for: Blood and Site of Disease Inflammatory Profiles Differ in Patients With Pericardial Tuberculosis and Human Immunodeficiency Virus Type 1
Source: Open Forum Infect Dis. 2023 Mar 9;10(3):ofad128. doi: 10.1093/ofid/ofad128 (PMC10043131; doi:10.1093/ofid/ofad128)
Supplement: ofad128_Supplementary_Data [file ofad128_supplementary_data.zip › Supplementary figures_20230220.docx]

**Supplementary fig. 1.** **Representative gating strategy for the phenotypic characterization of IFN-γ+CD4+ T cells. (a)** T cell lineage. **(b)** Mtb-300-specific CD4 response and HLA-DR expression.

**Supplementary fig. 2.** **Scatter plots of the 39 analytes detected in plasma of participants with LTBI, PTB and PCTB.** **(a)** Analytes that are significantly elevated in diseased groups compared to LTBI. **(b)** Analytes that were not expressed differently between the three groups. Statistical comparisons were performed using a Kruskal-Wallis test, adjusted for multiple comparisons (Dunn’s test).

**Supplementary fig. 3.** **Baseline levels of analytes detected in Plasma and PCF of participants with PCTB.** **(a)** Analytes elevated in PCF compared to Plasma. **(b)** Analytes elevated in Plasma compared to PCF. **(c)** Analytes that showed no difference between Plasma and PCF. Statistical comparisons were performed using a Wilcoxon test and p-values were adjusted using the Benjamini Hochberg multiple testing correction.

**Supplementary fig. 4.** **Univariate correlation of analytes detected in Plasma and PCF of participants with PCTB.** Analytes are arranged according to their correlation strength. Only analytes with significant associations are shown. ﻿The line indicates linear regression for statistically significant correlations. Correlations were tested by a two-tailed non-parametric Spearman rank test.

**Supplementary fig. 5.** **Longitudinal levels of analytes detected in plasma of participants with PTB and PCTB at Baseline, Week 6/8 post treatment initiation and at the end of treatment (Week 24). Week 24 was further compared and LTBI.** **(a)** Analytes that showed significant reduction with treatment in both PTB and PCTB. **(b)** Analytes that showed significant reduction with treatment in PTB only. Statistical comparisons were performed using a Friedman test, adjusted for multiple comparisons (Dunn’s test) for BL v W6/W8, BL v W24 and W6/W8 v W24 and the Mann-Whitney test to compare LTBI with W24, p-values were adjusted using the Benjamini Hochberg multiple testing correction.

**Supplementary fig. 6.** **Ability of HLA-DR expression on Mtb-specific CD4 T cells to discriminate LTBI from PTB, PCTB or any active TB (PTB + PCTB).** **(a)** Receiver operating characteristics (ROCs) curves for HLA-DR HLA-DR expression on Mtb-specific CD4 T cells in discriminating LTBI from PTB, LTBI from PCTB and LTBI from any active TB (PTB + PCTB), respectively. **(b)** Corresponding sensitivity and specificity for each ROC curve at the optimal threshold of HLA-DR expression on Mtb-specific CD4 T cells to distinguish between the groups.
